# Comparison of structure- and ligand-based scoring functions for deep generative models: a GPCR case study

**DOI:** 10.1186/s13321-021-00516-0

**Published:** 2021-05-13

**Authors:** Morgan Thomas, Robert T. Smith, Noel M. O’Boyle, Chris de Graaf, Andreas Bender

**Affiliations:** 1 Centre for Molecular Informatics, Department of Chemistry, University of Cambridge, Cambridge, CB2 1EW UK; 2 Computational Chemistry, Sosei Heptares, Steinmetz Building, Granta Park, Great Abington, Cambridge, CB21 6DG UK

**Keywords:** Artificial Intelligence, AI, Structure-based drug design, SBDD, Ligand-based drug design, LBDD, Deep learning, Generative models, Recurrent neural network, Molecular docking, Reinforcement learning, De novo design, Quantitative structure–activity relationship, QSAR

## Abstract

Deep generative models have shown the ability to devise both valid and novel chemistry, which could significantly accelerate the identification of bioactive compounds. Many current models, however, use molecular descriptors or ligand-based predictive methods to guide molecule generation towards a desirable property space. This restricts their application to relatively data-rich targets, neglecting those where little data is available to sufficiently train a predictor. Moreover, ligand-based approaches often bias molecule generation towards previously established chemical space, thereby limiting their ability to identify truly novel chemotypes. In this work, we assess the ability of using molecular docking via Glide—a structure-based approach—as a scoring function to guide the deep generative model REINVENT and compare model performance and behaviour to a ligand-based scoring function. Additionally, we modify the previously published MOSES benchmarking dataset to remove any induced bias towards non-protonatable groups. We also propose a new metric to measure dataset diversity, which is less confounded by the distribution of heavy atom count than the commonly used *internal diversity* metric. With respect to the main findings, we found that when optimizing the docking score against DRD2, the model improves predicted ligand affinity beyond that of known DRD2 active molecules. In addition, generated molecules occupy complementary chemical and physicochemical space compared to the ligand-based approach, and novel physicochemical space compared to known DRD2 active molecules. Furthermore, the structure-based approach learns to generate molecules that satisfy crucial residue interactions, which is information only available when taking protein structure into account. Overall, this work demonstrates the advantage of using molecular docking to guide de novo molecule generation over ligand-based predictors with respect to predicted affinity, novelty, and the ability to identify key interactions between ligand and protein target. Practically, this approach has applications in early hit generation campaigns to enrich a virtual library towards a particular target, and also in novelty-focused projects, where de novo molecule generation either has no prior ligand knowledge available or should not be biased by it.

**Supplementary Information:**

The online version contains supplementary material available at 10.1186/s13321-021-00516-0.

## Introduction

Generative models are a class of machine learning algorithms that model the distribution of training data, such that new data instances can be generated that resemble the training data distribution. These models have been successfully applied to de novo molecule generation, namely deep generative models which utilise deep neural networks [1–3]. Generative models for de novo molecule generation can generate valid and novel chemical structures [4] by either learning from a dataset of example molecules or learning appropriate actions to take given a set of symbolic rules. Although these models vary greatly in method [5], they can usually be categorised by four common architectures/approaches: (1) Language models (such as [6–8]) which require a chemical language (e.g., SMILES [9]) to represent molecular structure and subsequently learn the probability of a symbol in a sequence given all previously observed symbols; (2) Autoencoders (such as [10–12]) which use an encoder and decoder network to embed molecules into a fixed size latent space which can then be traversed to generate de novo molecules; (3) Generative adversarial networks (such as [13–15]) which use a generator and discriminator neural network to transform random noise into a distribution indistinguishable from real data and (4) Pure reinforcement learning (such as [16–18]) which uses neural networks to learn which actions to take given a set of molecule building rules.

The common goal of de novo molecule generation is to generate molecules within a desired property space, which in turn is often defined using ligand-based objectives or scoring functions. Examples include using known bioactive molecules as training data to bias generation towards a similar property space (e.g. biased training or fine-tuning) [2, 6, 19], or using machine learning models trained on known bioactive molecules to predict de novo molecule bioactivity (e.g. quantitative structure–activity relationship (QSAR) models). Generative models can then be optimized to maximize this predicted value e.g. using reinforcement learning [7, 8, 20, 21], Bayesian optimization [22] or particle swarm optimization [23]. Hence, a multitude of generative model methods exist, that can use none, one or multiple QSAR models or other external scoring functions to evaluate de novo molecules. Furthermore, generative models can then be optimized by one of several possible optimization algorithms. In drug discovery, most combinations of these methods rely on ligand data to optimize towards bioactivity.

However, ligand-based scoring functions (e.g. QSAR models) have inherent limitations. Firstly, machine learning models are restricted by their applicability domain i.e. they perform well on ‘in-distribution’ data but struggle to extrapolate to ‘out-of-distribution’ data, which is often poorly accounted for in model validation [24, 25]. This means that models will score molecules similar to those observed in the training data more accurately [26]. In fact, Renz et al. [27] recently demonstrated that deep generative models optimizing QSAR model predictions biased molecule generation towards these QSAR models so much that generated molecules were no longer predicted as active by control QSAR models, which were either initialized with a different seed or trained on a different data split. This showed that ligand-based scoring functions can subsequently lead to biased molecule generation, optimizing just one of many possible desirable property spaces—the one *most similar to training data* and *conforming to particular model parameters and hyperparameters*. This is very likely a reason for the lack of diversity (and inability to access truly novel chemical space) seen in deep generative models [28, 29]. This bias towards specific training data (either directly via fine-tuning or indirectly via ligand-based scoring functions) therefore restricts the novelty aspect of such ligand-guided deep generative models in practice and limits their exploration of novel chemical space. This is a serious drawback from both an intellectual property and discovery perspective; for example, during lead optimization the inability to discover novel chemistry can lead to property ‘dead zones’, where it can be difficult to optimize certain properties of a particular lead series further. This lack of novelty observed in current deep generative models has also been commented on in the literature [30]. Hence, the choice and implementation of ligand-based approaches can have a significant impact on de novo molecule generation.

From a practical perspective, ligand-based scoring functions also require large enough amounts of annotated ligand data to sufficiently train a machine learning model in the first instance, which typically restricts the use of machine learning models to data-rich areas. However, many key drug discovery objectives, such as being *first-in-class* with respect to a novel target, are typically ligand data poor. This, therefore, even conceptually prevents the application of ligand-guided deep generative models in this situation.

In this work, we explored the idea that structure-based scoring functions, as exemplified by molecular docking, may mitigate some of the limitations observed with ligand-based scoring functions. Molecular docking is a physics-based approach that uses the crystal structure (or in the absence of that a homology model) of a protein to estimate both the pose and free energy binding of a ligand [31–34]. Although the resulting free energy score is notoriously inaccurate [35, 36] and the performance of these scoring functions can be highly target-dependent [37], molecular docking consistently results in the early enrichment of known active molecules in virtual libraries compared to random [35] and is a generally-applied computational ligand design method in pharmaceutical research today.

The principal advantage of the physics-based nature of molecular docking is that it is not restricted to the chemical space of existing bioactive training data from the ligand side. Provided a scoring function achieves enrichment of bioactive compounds against a protein target (which can be established on existing datasets where data is available, but where otherwise estimates can be made based on the character of the binding pocket and protein type [38, 39]), then the chemical space to be scored can be greatly expanded, beyond chemistry and chemotypes present in any ligand-based training dataset. As structural input, either experimentally resolved structures or homology models can be employed and given the increasing numbers of structures available (which increases by about 10,000 per year [40]) and development of protein structure prediction technology [41], this renders this approach applicable to an increasingly wide range of protein targets.

In concrete terms regarding the methods employed, we utilized the REINVENT [7] algorithm that has evidenced competitive performance with respect to the coverage of de novo chemical space [42]. REINVENT uses a language-based generative model that takes in molecular SMILES as input (one-hot encoded) and a recurrent-neural network to predict the probability of the next SMILES symbol given all previously sampled SMILES symbols in a sequence. REINVENT uses reinforcement learning to optimize molecule generation to maximize a reward provided by an external scoring function (for further details see “Methods”). We used this approach to optimize de novo molecules to minimize the docking score returned by Glide [32]. To understand the differences between ligand-based and structure-based scoring functions, we compare the resulting de novo molecules to those generated by a model optimized to maximize the predicted probability of activity by a support vector machine (SVM) scoring function. This work could also be conducted using open-source docking software (e.g. Smina [43]) which we also provide available code for (see “Availability of data and materials”).

As a case study, we chose affinity for Dopamine Receptor D2 (DRD2). This receptor has a wealth of associated ligand bioactivity data available, and it has been commonly used in deep generative model publications before [7, 21, 22, 29, 44], thereby allowing any further comparison to different methods. DRD2 also has a publicly available X-ray crystal structure [45] in complex with Risperidone, thereby allowing use of molecular docking without the requirement of generating a homology model. More generally, G protein-coupled receptors (GPCRs)—including DRD2—are the most commonly targeted protein class accounting for approximately 34% of all FDA approved drugs [46]. However, they remain some of the most difficult proteins to crystallise. Although more structures are released every year [40], which offers an ever increasing opportunity to utilise structure-based design [47].

To our knowledge, few previous studies exist which have incorporated structural data into deep generative model scoring functions, compared to the ligand-based counterpart. Firstly, Ghanakota et al. [48] combined high throughput free energy perturbation (FEP) with REINVENT to identify potential CDK2 inhibitors. To achieve this, they trained an AutoQSAR model [49] on a subset of 1,000 enumerated analogues of a potent inhibitor with the corresponding FEP predictions, which was subsequently used as the REINVENT scoring function. The authors observed 1.5-fold enrichment selecting compounds with activity below 10 nM, compared to selecting enumerated analogues using the AutoQSAR model alone. Secondly, Li et al. [50] trained a recurrent neural network on known kinase CDK4 inhibitors and fine-tuned the network by training on a selection of generated molecules screened using docking. This was validated experimentally, with one out of nine tested molecules found to be active against the target (57.8% inhibition at 10 µM). Thirdly, Xu et al. [51] similarly used molecular docking to guide ligand selection in the latent space of a variational autoencoder towards CDK2 predicted activity, resulting in the recovery of a known CDK2 inhibitor and several molecules containing substructures of known CDK2 inhibitors. Cieplinksi et al. [52] evidenced that CVAE [10] and GVAE [53] were unable to generate molecules with optimized Smina [43] docking scores due to the inaccurate prediction of said docking score, which is used to guide de novo sampling in the respective methods. Although, the authors propose a docking benchmark on which REINVENT outperforms the above methods and baselines of both random and known active molecules [54]. Lastly, Boitreaud et al. [55] recently used a novel sampling approach combined with a graph to SELFIES [56] variational autoencoder, where the authors demonstrated the ability to optimize the Vina [34] docking score against Dopamine Receptor D3, while maintaining chemical diversity.

Notable contrasts in our approach compared to the above approaches include: (1) We only require structure data, enabling the search of a much larger chemical space compared to the use of ligand data as in [50]. (2) We utilize a recurrent neural network with reinforcement learning as opposed to a variational autoencoder with Bayesian optimization as in [55]. (3) We directly use a physics-based scoring function (i.e. molecular docking) to obtain scores during the generative model training process, as opposed to predicting the outcome of said function via machine learning as in [48, 52]. (4) In our approach, the model actively learns the conditional probability distribution of SMILES symbols that are associated with better docking scores and as such variable size distributions can be sampled (up to billions [57]) of molecules, as opposed to sampling a finite latent space as in [51]. Therefore, our approach presented here differs to previously published approaches conceptually.

## Methods

**Fig. 1:**
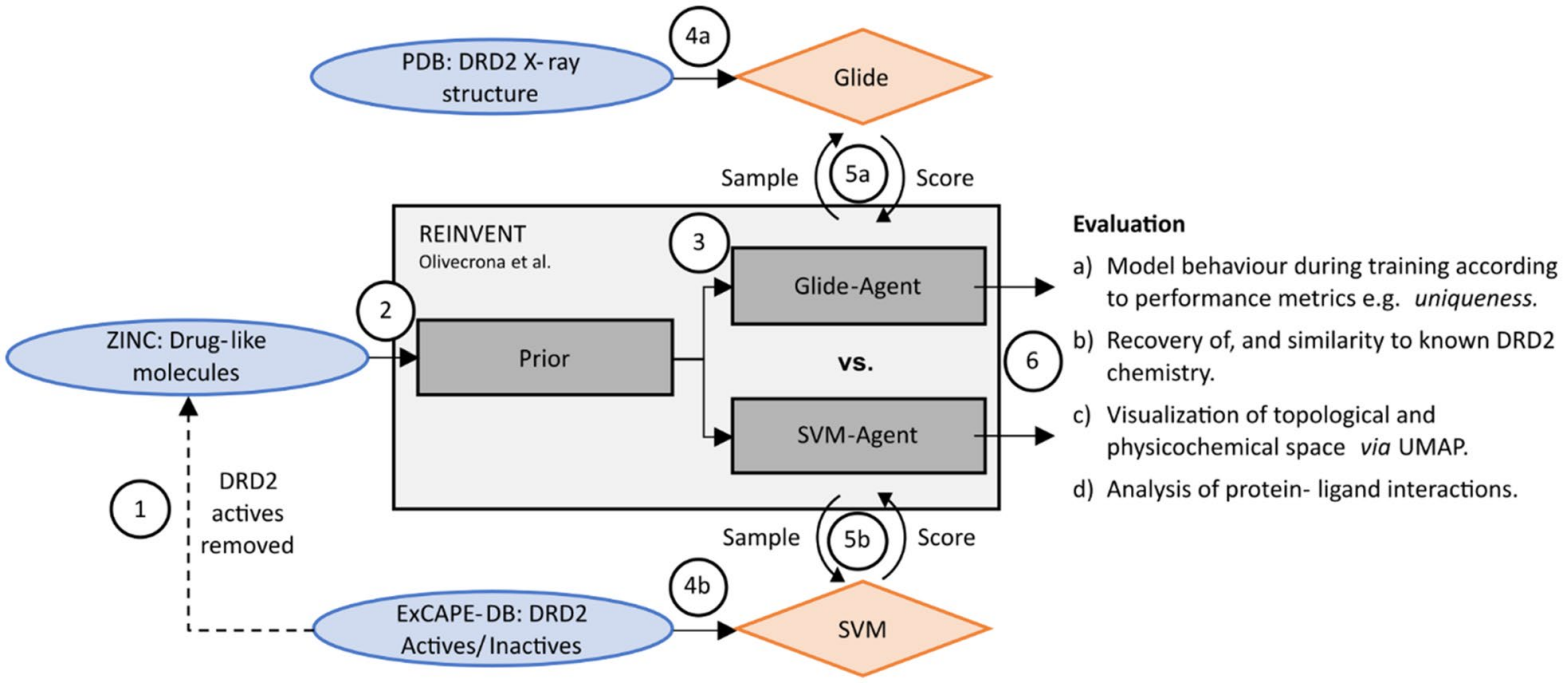
Schematic of this work including data sources (blue), scoring functions (orange), the deep generative model framework REINVENT [7] (grey). Main steps are (1) removing known DRD2 active molecules from the ZINC training data; (2) Training the Prior model on drug-like molecules from ZINC; (3) Initializing the Agents as a copy of the Prior; (4) Preparing the scoring functions to evaluate de novo molecules; (5) Iteratively training both Agents via reinforcement learning; and (6) evaluating the structure- and ligand-based approach with respect to different quantitative, chemical and structural aspects of the generated molecules

Figure 1 depicts the approach taken for the comparison of a structure- and ligand-based scoring functions in a deep generative model setting undertaken in this work. We (1) first removed known DRD2 actives (according to the ExCAPE-DB [58]) from the MOSES curated [4] ZINC [59] database of small drug-like molecules for use as training data. We then utilized the REINVENT framework [7] as a deep generative model. This framework consists of two recurrent neural networks—a Prior and an Agent. The Prior (2) is trained to learn the conditional probability distribution of symbols in one-hot encoded SMILES, in this case, a set of SMILES from the previously described ZINC training data. The Agent is then initialized (3) as an exact copy of the Prior. The scoring functions (4) used in this work either (4a) utilized structural data from the PBD [40] and the docking program Glide, or (4b) ligand data extracted from ExCAPE-DB [58] to build an SVM-based bioactivity model [7] to score molecules that have been generated de novo. The agent then samples de novo SMILES strings which are subsequently evaluated by the scoring function (5), and the Agent is updated via reinforcement learning to optimize either the docking score (5a) or the predicted probability of activity (5b). One unique aspect of REINVENT is the use of the Prior network to evaluate the likelihood of Agent de novo molecules being sampled from it, and this likelihood is used within the reward term used to update the Agent. This acts to both regularize the Agent to prevent overfitting, but also to ensure that the Agent does not forget the underlying chemical principles learned from the Prior training dataset. For more detail about REINVENT the reader is referred to the original publication [7]. Finally (6), we evaluated both model behaviour during Agent training and properties of de novo molecules with respect to several different quantitative, chemical and structural aspects.

### Datasets

The dataset used to train the Prior network was modified from the curation described by MOSES [4], in which the authors extracted molecules as SMILES from the ZINC15 database [59]. In short, molecules were selected to adhere to the following rules: molecular weight between 250–350 Da; number of rotatable bonds not greater than 8; XlogP [60] not greater than 3.5; no charged atoms; no atoms besides C, N, S, O, F, Cl, Br, H; no cycles larger than 8 members; custom medicinal chemistry filters [61, 62]; and finally PAINS filters [63] were applied. We deviate from this curation by first allowing charged atoms and then neutralizing protonatable groups. This was achieved by modifying the MOSES pipeline, implemented using RDKit [64], to remove the filter that checks for formal charge on atoms and instead add/remove protons to neutralize atoms where possible [65]. As a result, the training split contains 2,454,087 molecules as opposed to 1,584,664 described in the publication [4]. The authors rationalized the charge filter as such, “we removed charged molecules to avoid ambiguity with tautomer’s and pH conditions. Note that in the initial set of molecules, functional groups were present in both ionized and unionized forms”. However, given the nature of molecule generation conditional upon the rest of the molecule (or more specifically the sequence) using RNNs used in this work, we instead hypothesize that this filter could remove potentially relevant chemical structures in which the ‘functional group—whole structure’ conditional relationship may not be duplicated. We further find that only ~ 6500 charged variants are also present in the neutral form in the ZINC15 subset out of the ~ 870,000 removed due to the charge filter. This may further lead to a bias towards non-protonatable chemical structures which are crucial for aminergic receptors as used in this work, as aminergic receptors typically require an ionic interaction with a conserved aspartic acid residue in the orthosteric site (Ballesteros-Weinstein: D^3.32^, GPCRdb: D^3x32^) [66, 67]. To further require the deep generative model to explore novel chemical space, we also removed any canonical SMILES that matched the canonical SMILES of any known DRD2 active molecules extracted from the ExCAPE-DB [58] where canonical SMILES were generated using RDKit [64] for both sets. This resulted in a training set of 2,454,048 canonical SMILES.

In order to generate a set of bioactive compounds with known DRD2 activity we extracted molecules from ExCAPE-DB [58]. ExCAPE-DB is a curation of ChEMBL20 [68] and PubChem [69] data that classifies molecules with a measured dose–response value equal to or lower than 10 μM as active, and with higher than 10 μM (or those which were labelled inactive in the original sources) as inactive. This resulted in 4613 active and 343,028 inactive molecules against human DRD2. However, as it may be unreasonable to expect the generative model to generate molecules outside the property space on which it was trained, we also apply the same filtering as previously described to create another subset labelled ‘in’. In addition, for use as a reference baseline a set of random molecules with the same filters applied were extracted from ChEMBL26 [68]. Resulting in the following subsets (of size): Active_all (4613), Active_in (396), Inactive_all (10,000), Inactive_in (10,000), Random (10,000).

The DRD2 X-ray crystal structure 6CM4 from the PDB [40] was used as the protein structure for docking.

### Reinvent

The training data described in Datasets was subject to further filtering in accordance with the REINVENT pipeline [7] to standardize SMILES input, tokenize SMILES symbols and construct a vocabulary for one-hot encoding. This filtering resulted in 2,453,916 unique, non-isomeric (stereochemistry removed) SMILES that was subsequently used to train the Prior network for a total of 5 epochs with a batch size of 128 using the Adam optimizer [70] with a learning rate of 0.001. The Agent was then trained for 3000 steps using a batch size of 64 and the Adam optimizer with a learning rate of 0.0005 and a value for the scalar coefficient (σ) of 60. These hyperparameters were used as recommended by the publication [7] and not explored further. All neural network training was conducted on an NVIDIA RTX2080_Ti_ GPU.

### Scoring functions

A ligand-based scoring function was used as a baseline. We used the SVM model previously published by Olivecrona et al. [7] trained on 7218 active and 100,000 inactive DRD2 molecules, which were also extracted from ExCAPE-DB [58]. Note that this figure differs from the human DRD2 bioactives we used for evaluation described in Datasets for the current work. It is likely that the authors did not filter bioactive molecules by species (as it stands this would result in 7919 active DRD2 molecules without further processing [58]), which however is particularly important in the current work due to the use of the human ortholog of DRD2 for docking, and hence we have paid particular attention to this here. The resulting SVM predicts the uncalibrated probability of a molecule to be active against DRD2.

The structure-based scoring function used protein–ligand docking. The DRD2 crystal structure was prepared using the Schrodinger Protein Preparation Wizard [71] using default parameters i.e. we added hydrogens, protonated non-residue molecules (e.g. ligand, cofactors), at pH 7 ± 2 using Epik [72], optimized hydrogen bond assignment at pH 7 using PROPKA [73] and minimized the structure using the OPLS3e force field [74]. Any waters, cofactors, or crystallisation artefacts (e.g., oleic acid) were removed from the structure. A grid was defined using the centroid of the co-crystallised ligand Risperidone as the centre. From the ligand side, before docking, molecules were prepared using LigPrep [75], enumerating unspecified stereocentres, tautomers and protonation states (using Epik [72]). Up to 8 variants were prepared per molecule based on a pH range of 7 ± 1 and minimised using the OPLS3e force field. Each molecule and any respective variants were then docked using Glide standard precision (GlideScore-SP [32]) with default settings, flexible ligand sampling, standard precision with Epik state penalties, post-docking minimization of five poses and final output of the single best scoring pose. For molecules where more than a single variant exists, the variant with the lowest (best) docking score was chosen. To make this task more computationally tractable, we used a Python script that parallelized the docking protocol across a compute cluster using the python library Dask [76]. Using between 36 and 50 CPUs, the wall time required for 3000 iterations was approximately 7 days, based on an average scoring time of 3 min per 64 molecules (including molecule preparation and up to 512 individual docking runs for respective variants).

### Retrospective validation of docking protocol and scoring functions

In the REINVENT study [6] the authors evaluated the performance of the SVM model on an undisclosed held-out test set, resulting in an accuracy of 98%, precision of 97% and recall of 82%.

To also evaluate the performance of the docking protocol, all 4613 known DRD2 active molecules and a random subset of 10,000 DRD2 inactive molecules were docked. The performance of classification into either active or inactive molecules at various docking score thresholds was then investigated (see Additional file 1: Figure S1) according to classification accuracy, precision, and recall (which can be calculated using the equations defined below and the number of *true positives (TP), true negatives (TN), false positives (FP) and false negatives (FN)*). A docking score of − 7.5 resulted in highest overall accuracy of about 76%. By decreasing the threshold to − 8.5 (i.e., a more stringent criterion for selecting active molecules), a higher precision of approximately 82% is achieved, although at lower accuracy of about 74% and lower recall of about 12%. However, the latter more stringent threshold might still be a more favourable one to use in practice, given that confidence in positive predictions of active compounds is often more relevant than missing some active compounds (of which there are many) due to low recall. It should be remembered that the performance of the scoring function was not an objective in its own right (given that retrospective evaluations naturally favour ligand-based methods due to analogue bias in databases etc. [77]), but rather to ensure general suitability for the desired purpose of selecting active compounds in this step.

$$Accuracy = \frac{TP + TN}{{TP + FP + TN + TN}}$$

$$Precision = \frac{TP}{{TP + FP}}$$

$$Recall = \frac{TP}{{TP + FN}}$$

### Model performance and diversity metrics

Several metrics were used to assess generative model performance, as used in GuacaMol [78] and MOSES [4] (see Additional file 1).

In particular, we propose a new metric to measure the diversity of de novo compounds which we call *sphere exclusion diversity (SEDiv). SEDiv* is the fraction of diverse compounds selected using the sphere exclusion algorithm [79] with a sphere radius set to 0.65 Tanimoto distance of Morgan fingerprints (*radius* = *2, nBits* = *1024*), using the algorithm implemented by Roger Sayle in RDKit [64, 80]. We interpret this as the minimum fraction of the dataset required to explain the chemical diversity in the context of bioactivity. As a set distance threshold of 0.65 (i.e., Tanimoto similarity of 0.35 or above) broadly correlates to an 80–85% probability of belonging to the same bioactivity class [80].

As opposed to *internal diversity* (see Additional file 1)*,* we believe the interpretation of this metric to be more meaningful. As the *internal diversity* can be difficult to interpret due to the double average losing the notion of the underlying distribution, as well as the confounding effect of heavy atom count on Tanimoto similarity [81]. To investigate this further, we subset ChEMBL28 [82] to only include molecules with 5–50 heavy atoms and randomly sampled 500 molecules either side of a heavy atom threshold, for thresholds 10–45 in increments of 1 (with 10 repeats per threshold)—to mimic datasets with different proportions of smaller/larger molecules. There is a clear decrease in *internal diversity* with an increase in mean number of heavy atoms in accordance with the hypothesized confounding effect [81] (see Additional file 1: Figure S2a). On the other hand, *sphere exclusion diversity* shows a similar trend to the count of molecules per heavy atom bin (see Additional file 1: Figure S2b).

**Fig. 2:**
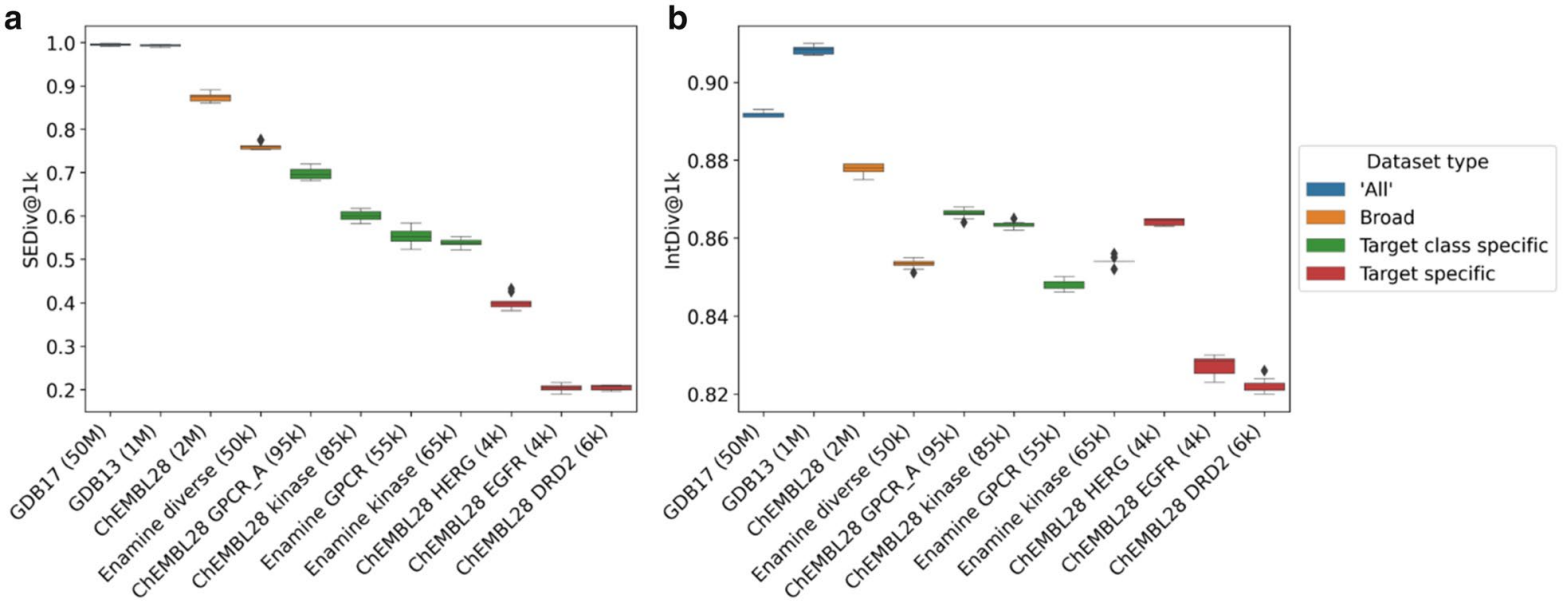
The measured *sphere exclusion diversity (SEDiv)* (**a**) and *internal diversity (IntDiv)* (**b**) of a randomly sampled 1000 (@1k) subset of a variety of virtual libraries and datasets of characterised molecules with activity against particular targets belonging to a target class, or single targets. *Internal diversity* shows counterintuitive behaviour such as, measuring GDB13 as more diverse than GDB17 and hERG active molecules as diverse as molecules active against any family A GPCR, any kinase or a virtual library designed towards achieving diversity. Conversely, *sphere exclusion diversity* measures diversity in line with chemical intuition

To investigate the difference between *SEDiv* and *internal diversity* further, we calculate these two metrics on random subsets of different libraries (Fig. 2): enumerated virtual libraries of stable molecules up to 17 and 13 heavy atoms (GDB17 [83], GDB13 [84]), characterised molecules with varying bioactivities (ChEMBL28 [82]), a synthetically accessible diversity orientated virtual library (Enamine diverse [85]), synthetically accessible targeted virtual libraries (Enamine GPCR and Enamine Kinase [86]) and characterised molecules with activity (pChEMBL ≥ 5) against specific target classes (ChEMBL28 Family A GPCRs and ChEMBL28 Kinases) and single targets (ChEMBL28 HERG, ChEMBL28 EGFR and ChEMBL28 DRD2). All datasets were similarly processed to neutralize molecules and retain only those with a molecular weight less than 500 Da, to ensure a similar ‘drug-like’ chemical space. Most notably, *internal diversity* measures GDB13 as more diverse than GDB17—which contradicts chemical intuition, but further confers with hypothesized confounding effects [81]. Furthermore, *internal diversity* measures molecules active against hERG—a promiscuous target related to cardiotoxicity [87]—as diverse as all molecules reported active against any kinases, any family A GPCR and more diverse than a virtual library designed for diversity. Conversely, *sphere exclusion diversity* measures GDB17 as more diverse than GDB13 (which is better distinguished at larger sample sizes, see Additional file 1: Figure S3) and hERG active molecules as more diverse than single targets (EGFR and DRD2) but not as diverse as all molecules active against any family A GPCR or kinase. Therefore, the proposed approach better aligns with chemical intuition regarding the chemical diversity of known libraries. Furthermore, this approach yields values in the full range of possible values 0–1 (unlike *internal diversity* which mostly lie in a range of ~ 0.7–0.9), which further has a direct interpretation as the fraction required to explain the chemical space; therefore, a comparative reference is not *always* necessary (unlike *internal diversity*). However, the values measured here provide some context for sample sizes of 1000 random molecules, which we recommend for future use in comparing de novo molecule diversity. Code to calculate the *sphere exclusion diversity* can be found at our associated GitHub page (see “Availability of data and materials”).

### DRD2 fingerprint analogues

Further to performance metrics, we also assess the number of molecular fingerprint analogues generated to known DRD2 active molecules. We follow similar methods as used in [29], converting molecules to Morgan fingerprints (*radius* = *2*, *nBits* = 1024), where analogues were considered to be two molecules with a fingerprint Tanimoto similarity greater than or equal to 0.4. As opposed to [29], we used a smaller Morgan fingerprint radius and bit length in line with the other metrics used in this work, and did not require molecules to have a particular predicted probability of DRD2 activity, as predicted by the SVM.

### Clustering

Molecular clustering was performed on molecules or their respective Bemis-Murcko scaffolds [88] using the sphere exclusion algorithm [79] as implemented by Roger Sayle [80] in RDKit [64]. The sphere radius was set at a Tanimoto distance of 0.65 and 0.2 for molecules or their respective scaffolds using Morgan fingerprints (*radius* = *2, nBits* = *1024*). Once resulting sphere centroids had been picked, molecules were assigned to the nearest centroid to form a cluster.

### Chemical space visualization

In order to further understand the chemistry generated by both approaches (and their distribution across chemical space), Uniform Manifold Approximation and Projection (UMAP) [89] was performed using both molecular fingerprint and physicochemical/property space representations, as well as calculating the normalized principal moments ratio (NPR) [90]. For the former, Morgan fingerprints (*radius* = 2, *nBits* = 1024, implemented using RDKit) of actives (either ‘in’ or ‘all’), Prior, Glide-Agent and SVM-Agent molecules were used as input features, and the UMAP was calculated using the Jaccard distance metric with a minimum distance 0. For property space, the *MolLogP, MolWt, HeavyAtomCount, NumHAcceptors, NumHDonors, NumHeteroatoms, NumRotatableBonds, NumAromaticRings, NumAliphaticRings, RingCount, TPSA, FractionCSP3, QED* [91] and *SAscore* [92] were calculated using RDKit and scaled before input to UMAP using default parameters. Lastly, the *NPR1* and *NPR2* were calculated using RDKit after first generating 3D conformations using the ETKDG method [93].

### Structure interaction fingerprints (SIFts)

Structure Interaction Fingerprints (SIFts) [94] were calculated on all resulting docked poses in order to understand ligand–protein interactions available to the generated ligands. This resulted in a 9-element bit vector for each protein residue, corresponding to non-exclusive residue interactions. For simplification, we converted the non-exclusive 9-element bit vector (comprising the possible interactions *any contact, backbone, sidechain, polar, hydrophobic, hydrogen bond acceptor, hydrogen bond donor, aromatic, charged*) to exclusive residue interactions in a hierarchical manner according to the following order: *charged hydrogen bond donor/acceptor*, *hydrogen bond donor/acceptor*, *charged*, *aromatic*, *hydrophobic/polar*. For example, a residue initially defined as having *sidechain*, *polar*, *charged* and *hydrogen bond acceptor* interactions would be converted to *charged hydrogen bond acceptor*, due to this interaction type taking precedent in the above order. This simplification was performed to allow for more interpretable (and less redundant) subsequent analysis of the interactions observed.

## Results and discussion

### Optimization of SVM- and Glide-Agent-based scores by molecules generated de novo

**Fig. 3:**
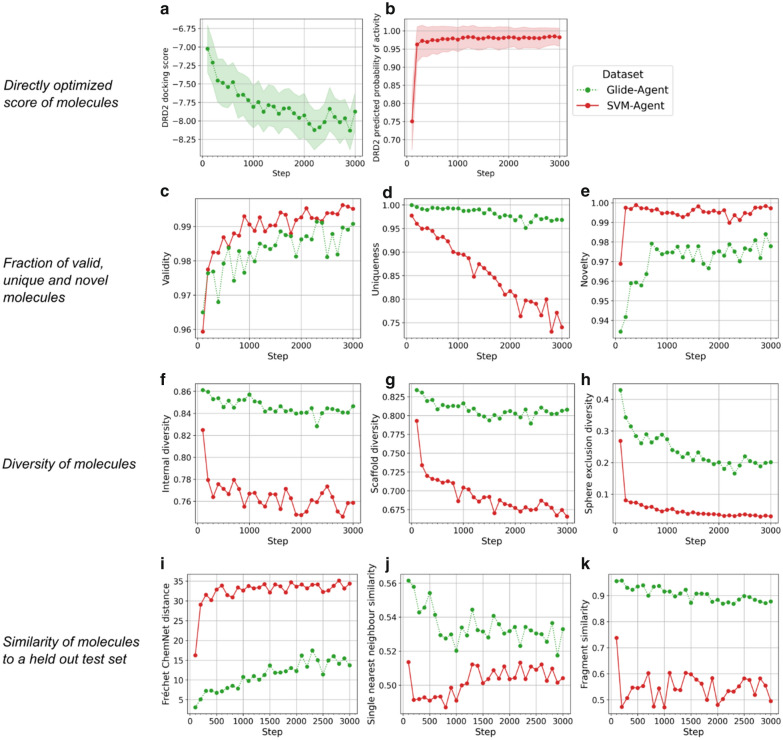
Generative model performance during optimization for the Glide-Agent (green) and the SVM-Agent (red), calculated every 100 steps. Mean optimization of scores—docking score and predicted probability of activity—are shown in (**a**) and (**b**) respectively, as well as the 95% confidence interval. Additional metrics shown are (**c**) *validity*, (**d**) *uniqueness*, (**e**) *novelty*, (**f**) *internal diversity*, (**g**) *scaffold diversity*, (**h**) *sphere exclusion diversity*, (**i**) *Fréchet ChemNet Distance*, (**j**) *single nearest neighbour similarity* and (**k**) *fragment similarity*. As the most important observation, the SVM-Agent reaches very high scores much more quickly, which comes at the cost of a significant reduction in uniqueness and diversity, when compared to the Glide-Agent. For definitions and detailed discussion see main text

We investigated whether the Agents were able to optimize the respective properties evaluated by the two scoring functions i.e.*,* predicted probability of DRD2 activity based on bioactivity data (‘SVM-Agent’) and DRD2 docking score (‘Glide-Agent’), the results of which are shown in Fig. 3. Both the SVM-Agent and Glide-Agent learn to generate molecules with optimized properties, albeit at different rates (Fig. 3a and b). Whilst the SVM-Agent converges to generating optimal molecules within just a few hundred steps, the Glide-Agent only begins to converge after about 2,000 training steps. Crucially, both Agents maintain high ratios of valid (> 0.9, Fig. 3c) and novel molecules per batch (> 0.9, Fig. 3e). However, from just 100 steps onwards, the SVM-Agent starts to generate fewer unique molecules than the Glide-Agent (Fig. 3d). This suggests overfitting, as the SVM-Agent has maximally optimized the scoring function and begins to re-sample molecules that it knows produce a high reward. This is further supported by a drop in the diversity of sampled molecules and their scaffolds (Fig. 3f–h). We also introduce a new diversity metric, *sphere exclusion diversity* (see “Methods”), which indicates that after 200 steps the chemical space of SVM-Agent de novo molecules can be explained by less than 10% of the *valid* and *unique* molecules, while for the Glide-Agent this slowly drops to about 20%. In addition, the SVM-Agent shows an increased FCD [95] to a held out test set with respect to the Glide-Agent (Fig. 3i). This increase in FCD has shown to indicate a number of differences [95] to the Prior training data for example, ‘drug-likeness’ defined by [91] or *internal diversity* [96]. Beyond the performance according to benchmark metrics, and similar to Blaschke et al. [29], we investigated the cumulative number of analogues generated de novo to known DRD2 active molecules (see Additional file 1: Figure S4). This analysis shows that the SVM-Agent generates more analogues (~ 80,000) than the Glide-Agent (~ 25,000), however, when instead looking at the number of DRD2 active molecules with generated analogues, the Glide-Agent has analogues to more DRD2 actives (~ 1800) than the SVM-Agent (~ 1400). Thus, the SVM-Agent generates more analogues per known active, but the Glide-Agent generates analogues to a broader range of known actives. Together, these results indicate that the Glide-Agent maintains better generative metrics throughout training, in particular with respect to the *uniqueness* and general diversity of the generated molecules. Also, the Glide-Agent generates analogues to more known DRD2 active molecules, further evidencing increased diversity with respect to known DRD2 active molecules.

**Fig. 4:**
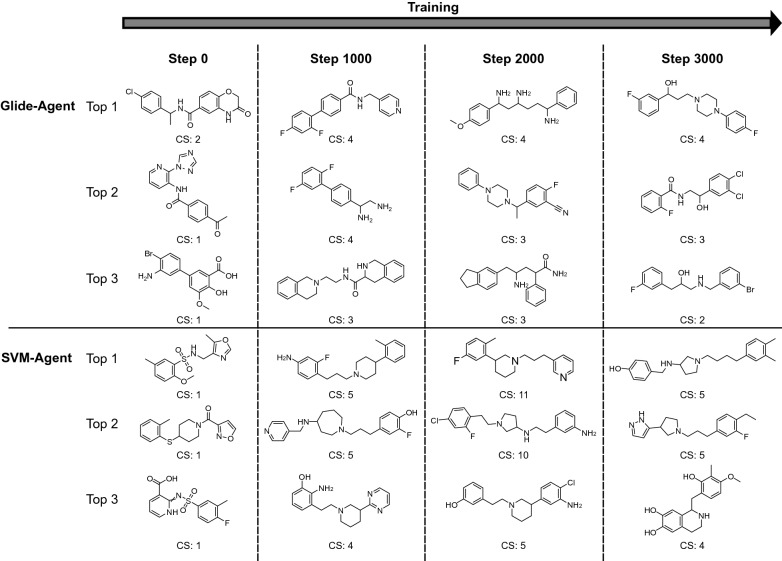
Chemotype evolution during training, comparing the SVM-Agent and the Glide-Agent. Molecules were sampled during training at the start and after 0, 1000, 2000 and 3000 steps (at the end of training). Molecules in each batch were clustered and the centroids of the three largest clusters are shown here, alongside respective cluster size (CS). This visualizes the difference in topology and chemotype between the two approaches

For any generative model, visual inspection of the generated molecules is crucial, both to see whether an approach tends to prefer different types of chemistry, and to identify any possibly idiosyncratic behaviour. In this regard, Fig. 4 displays the centroid of the largest clusters generated during training, as well as the respective cluster size. This shows that the chemotypes evolve from the Prior differently depending on the scoring function. Overall, both Agents were able to optimize molecules towards their respective scoring functions (as shown quantitatively in Fig. 3); however, the Glide-Agent does so with more diversity (Fig. 3f–h) and with a more similar distribution to the training data (Fig. 3i–k).

For further analysis, 10,000 molecules were sampled from the unoptimized Prior, the SVM-Agent (trained for 500 steps, before significant overfitting occurred), and the Glide-Agent (trained for 2000 steps). We calculated the suite of MOSES metrics [4] on the generated molecules (see Additional file 1: Table S1–S3) as well as, *Scaffold diversity* and *Scaffold uniqueness and Sphere exclusion diversity* (see “Methods”). Coinciding with the results observed in Fig. 3, the Glide-Agent outperforms the SVM-Agent in all metrics except *Novelty*. Overall showing greater diversity of de novo molecules and similarity to the training data (whilst still optimizing the docking score).

**Fig. 5:**
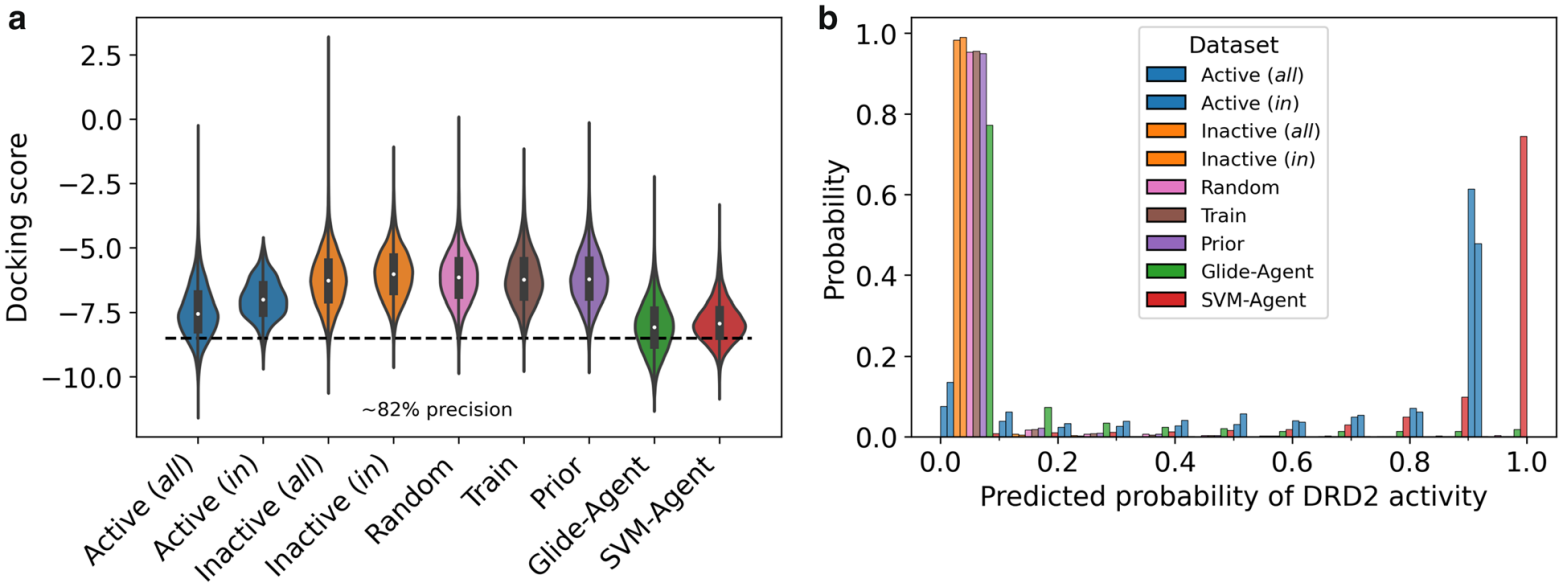
Docking scores (**a**) and predicted probability of DRD2 activity (**b**) of molecules generated de novo using the Prior, the SVM-Agent and the Glide-Agent, compared to the active, inactive, and random reference datasets. The more negative the docking score, the better it is predicted to bind. The Glide-Agent generated molecules have the best docking score distribution, more so than known DRD2 active molecules, whilst the SVM-Agent generated molecule distribution is more similar to known DRD2 active molecules. The SVM-Agent molecules and known DRD2 actives score most highly according to the SVM, comparatively, the Glide-Agent molecules do not

Next, we sought to better understand the extent to which the docking score could be optimized using our protocol, relative to known DRD2 active molecules. All molecules were docked, and their docking scores compared to the active, inactive and random reference dataset, the results of which are shown in Fig. 5a. The actives and inactives are further split into *‘all’* molecules extracted from ExCAPE-DB and molecules *‘in’* a similar chemical space as imposed by the same filters applied to the training data. The docking score distribution of the Glide-Agent de novo molecules (μ = − 8.05, σ = 0.95) is significantly enriched (one-tail t-test: adjusted p < 0.05) over unoptimized Prior molecules (μ = − 6.17, σ = 1.02) and importantly also over previously known DRD2 active molecules (μ = -7.45, σ = 1.01)(one-tail t-test: adjusted p < 0.05), especially those after filtering to impose similar chemical space restrictions (μ = − 6.96, σ = 0.74)(one-tail t-test: adjusted p < 0.05). In other words, the Glide-Agent de novo molecules are predicted to be often as active, and on average even more active, than known DRD2 active molecules according to the Glide docking protocol. If the precision for selecting active molecules for retrospective docking at a score threshold of − 8.5 (see “Methods”) translates also prospectively to de novo generated molecules, 32.70% percent of the Glide-Agent de novo molecules are predicted to be active against DRD2 (that is with a dose–response value lower than 10 µM), compared to 19.98% percent of SVM-Agent de novo molecules and 0.54% percent of Prior de novo molecules (which is relatively close to experimental hit rates that would be expected by chance alone, e.g. [97] which had an experimental hit rate against DRD2 of ~0.6%). Interestingly, the SVM-Agent de novo molecules also exhibit a significant enrichment (one-tail t-test: adjusted p < 0.05) in docking score distribution (μ = − 7.85, σ = 0.80) beyond known DRD2 active molecules, although to a lesser extent. This docking score distribution enrichment is hypothesized to be a factor of generating similar de novo chemistry to known DRD2 actives and hence, a docking score enrichment is observed. However, the improvement over known actives seen in Fig. 5a may also be due to an element of randomness, as Renz et al. observed different chemical space occupation for independent runs with similar models [27]. Furthermore, a previous run we conducted resulted in a smaller enrichment for the SVM-Agent but an almost identical enrichment for the Glide-Agent (data not shown). We also compared the predicted probability of DRD2 activity according the SVM (Fig. 5b) for all reference datasets. This shows that most known DRD2 actives and the SVM-Agent de novo molecules are predicted active with high probability (0.9–1.0). Unlike docking, which predicts SVM-Agent molecules to be equally as, or more active than known DRD2 active molecules, the SVM does not predict many Glide-Agent molecules to be active (about 75% with a low predicted probability of 0–0.1). Due to the limitations of such machine learning models discussed in the *Introduction,* we believe this could be evidence of a limited applicability domain. This is supported by the greater single nearest neighbour similarity of the SVM-Agent de novo molecules to DRD2 actives that were used by train the SVM model by [7] (see Fig. 7 and Additional file 1: Figure S5). Overall, we can conclude that the docking score of de novo molecules can generally be optimized by our Glide-based agent, and this is true even beyond the scores of known active molecules.

### Overlap analysis of molecules generated de novo compared to known active and inactive molecules

To assess recovery of known active molecules we identified whether any of the canonical SMILES produced by either Agent matches those of known DRD2 active molecules. The number of recovered molecules across ten samples of 10,000 molecules was converted into the probability of recovery (see Table 1) (based on valid and unique molecules generated). It is worth noting that the Prior has an inherent bias towards generating inactive molecules over active molecules, where we quantify the bias simply as the probability of generating a known active molecule over the probability of generating a known inactive molecule. This translates as the Prior being 0.002 times as likely to generate an active molecule compared to an inactive (which is partly also due to removing known DRD2 active molecules from the training data). When considering recovery of *‘all’* extracted DRD2 actives and inactives, both Agents are still biased towards generating inactive molecules; however, the Prior bias is improved 95-fold towards generating active molecules by the SVM-Agent. This bias shift is predominantly attributable to the SVM-Agent’s ability to *avoid* recovering known inactive molecules (approx. half the probability than the Glide-Agent), whereas the probability of recovering known active molecules is more comparable between the Glide- and SVM-Agents (63 × 10^–6^ vs 79 × 10^–6^, respectively). It is important to consider that Glide docking does not incorporate any prior knowledge of known DRD2 active and inactive molecules (unlike the SVM), and therefore the Glide-Agent is able to learn to recover known active molecules (and improve the Prior bias 40-fold) from the information of the scoring function alone. Interestingly, of the single sample of 10,000 molecules investigated throughout this work, there are no recovered active molecules in common between the Agents, and just three in total (see Additional file 1: Figure S6), further underlining their divergent behaviour. In summary, both Agents can similarly recover known DRD2 active molecules, however, the SVM-Agent is better at *not generating* known inactives and thus provide different types of molecules generated de novo as a result.

**Table 1:** Probability of recovering known DRD2 active and inactive molecules

Origin of dataset	Probability of generating active molecule (× 10^−6^)		Probability of generating inactive molecule (× 10^−6^)		Active bias (fold change from Prior)	
Active DRD2 chemical space relative to training data	In	All	In	All	In	All
Prior	10 (30)	10 (30)	5055 (604)	5957 (495)	0.002 (1)	0.002 (1)
Glide-Agent	11 (32)	63 (84)	422 (125)	917 (175)	0.025 (12.5)	0.069 (40.6)
SVM-Agent	34 (72)	79 (72)	256 (124)	486 (168)	0.130 (64.9)	0.163 (95.7)

The reported probability values are the mean (and standard deviation) across ten samples of 10,000 de novo molecules drawn from the model. The Glide- and SVM-Agent have a similar probability of recovering known active molecules, therefore the SVM-Agent bias towards generating active molecules over inactivate molecules is mostly driven by the lower probability of generating inactive molecules

### Similarity analysis of molecules generated de novo to known active and inactive molecules

We first repeated the analysis conducted during training, investigating the number of analogues to known DRD2 active compounds as in [29]. Similar to the results observed during training, Table 2 shows the SVM-Agent sample contains a higher fraction of molecules considered fingerprint analogues to DRD2 actives (both to actives *‘in’* a similar chemical space and *‘all’* extracted). Furthermore, both Agent samples contain a higher fraction of analogues to DRD2 actives than inactive molecules (which one would expect to be relatively high based on the chemical series nature of drug design). Although the Glide-Agent generates analogues to a higher fraction of DRD2 actives, indicating that the higher diversity observed (see Additional file 1: Table S2) is relevant with respect to active chemistry. In addition, the DRD2 actives with analogues generated differed depending on the Prior or Agent (see Additional file 1: Figure S7), evidencing complementary behaviour with respect to identifying similar molecules to known actives.

**Table 2:** Fraction of molecules that are fingerprint analogues to DRD2 active molecules and respective fraction of DRD2 active molecules with analogues

Origin of dataset	Fraction of molecules that are analogues to DRD2 actives		Fraction of DRD2 actives with analogues	
DRD2 chemical space relative to training data	In	All	In	All
Inactive *(in)*	0.020	0.089	0.197	0.116
Inactive *(all)*	0.025	0.102	0.242	0.116
Train	0.020	0.071	0.225	0.109
Random	0.024	0.075	0.313	0.120
Prior	0.021	0.071	0.220	0.110
Glide-Agent	0.051	0.124	0.268	0.105
SVM-Agent	0.179	0.563	0.237	0.102

The SVM-Agent generates more analogues to known actives, however, the Glide-Agent generates analogues to more known actives, demonstrating a greater coverage of known active space

**Fig. 6:**
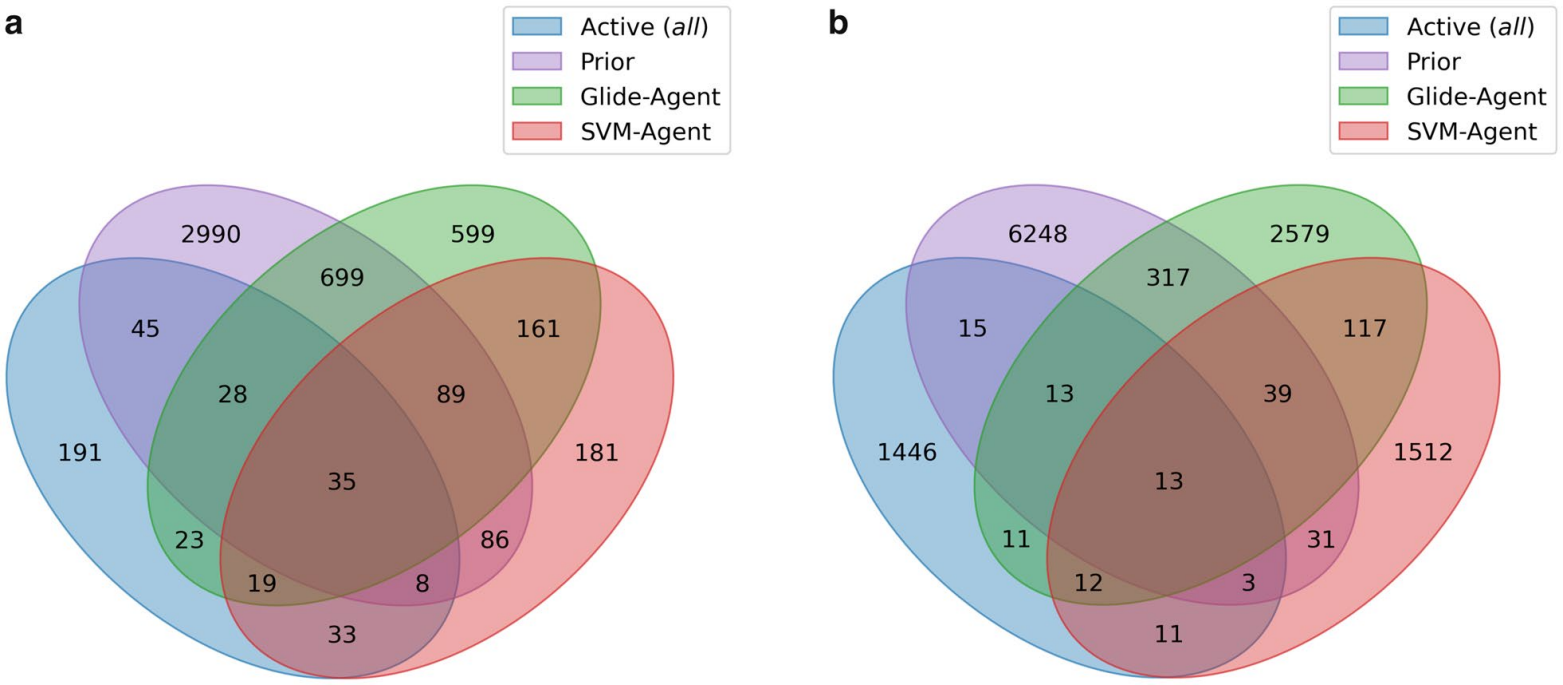
Chemical space overlap between the Prior, SVM- and Glide-Agents with all DRD2 ligands extracted from ExCAPE-DB. Broader clusters (**a**) were defined by clustering molecules with a Morgan fingerprint Tanimoto similarity to a centroid of 0.35 or greater, while narrower clusters (**b**) were defined by clustering molecules on their Bemis-Murcko scaffold Morgan fingerprint Tanimoto similarity to a centroid of 0.8 or greater (examples shown in Additional file 1: Figure S8-9). Numbers specify the number of clusters with at least one member belonging to an annotated dataset. For example, there are 23 clusters (**a**) where each cluster has at least one member belonging to DRD2 actives and Glide-Agent molecules. Both the Glide-Agent and SVM-Agent share clusters with known DRD2 active molecules

We also investigated how similar the de novo generated molecules were to known DRD2 active molecules and/or each other. The known DRD2 active molecules were clustered together with the Prior, Glide- and SVM-Agent de novo molecules. Each cluster was then analysed to identify to which dataset each of its members belonged (similar to [98]). Figure 6 shows the results of this analysis as a Venn diagram for both entire molecules (Fig. 6a) and their respective Bemis-Murcko scaffolds (Fig. 6b). This analysis shows more clusters-105—are shared between known active DRD2 molecules and the Glide-Agent, compared to the overlap of known active DRD2 ligands with the SVM-Agent, where this number is 95. This is also observed when clusters are calculated based on scaffolds (49 vs 39 respectively). To qualitatively assess cluster behaviour, examples of clusters and structures are shown in Additional file 1: Figure S8–S9. Overall, both the Glide-Agent and SVM-Agent share a relatively similar number of clusters (i.e. ‘chemical space pockets’) with known DRD2 actives, but which precise clusters are shared differs largely between both Agents.

### Novelty of de novo molecules relative to known DRD2 active molecules

**Fig. 7:**
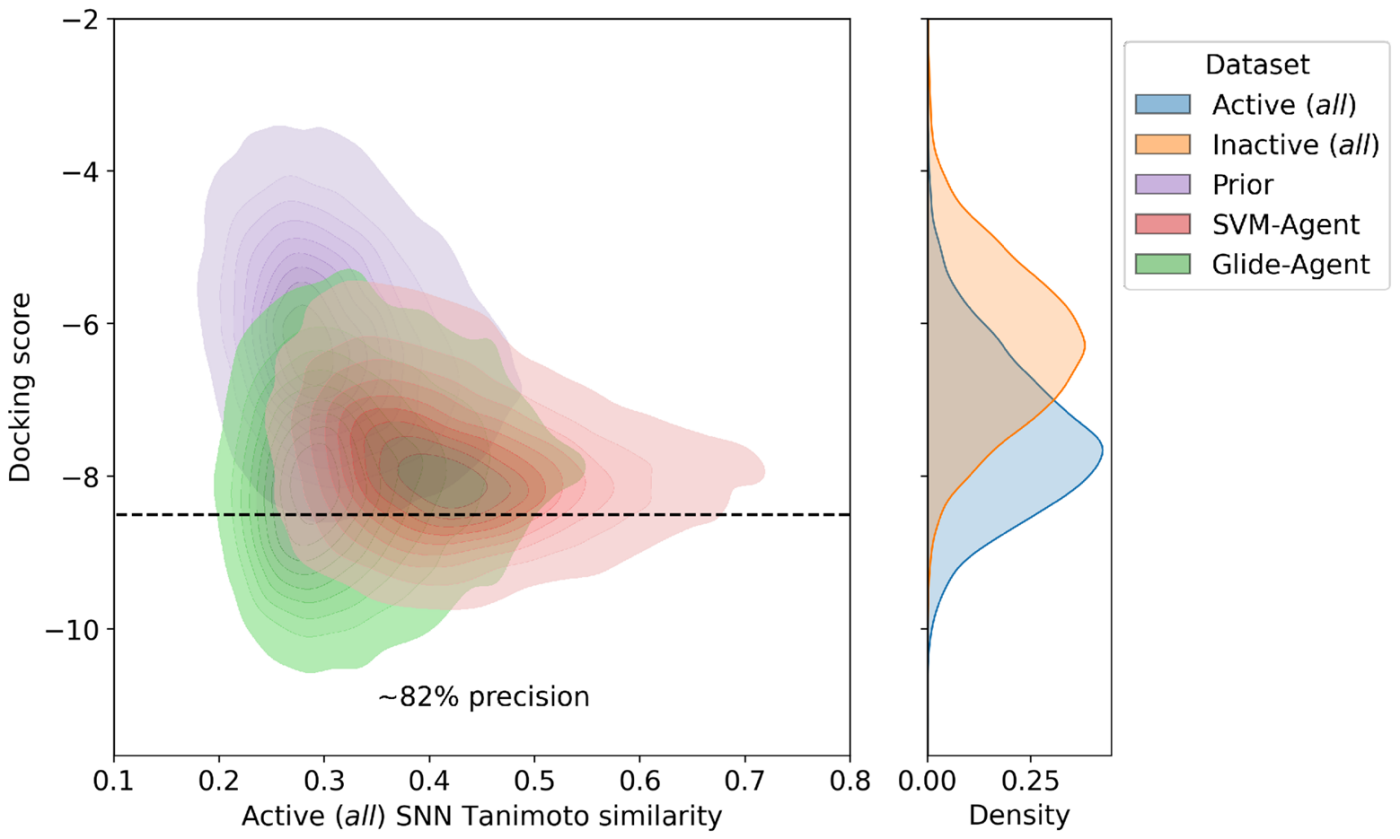
Kernel density estimates of the bivariate distribution of docking score and *single nearest neighbour similarity* to known DRD2 active molecules. The Glide-Agent distribution contains a shoulder with lower (better) docking scores at lower similarity to known actives than the SVM-Agent and Prior de novo molecules

Similarity comparisons of de novo molecules to known molecules with desirable properties can provide a measure of confidence that a model is in the correct chemical space. However, prospective use case ultimately requires structural novelty to known compounds with activity against the same biological target. Figure 7 shows that the Glide-Agent generated molecules that have enriched docking scores below the retrospective threshold of − 8.5 also have lower *single nearest neighbour similarity* to known DRD2 active molecules than the SVM-Agent and Prior molecules. Therefore, the Glide-Agent molecules are not only predicted more active but are also more novel with respect to known actives than the SVM-Agent molecules. This could prove very important in the early stages of hit discovery.

### Differences in chemical substructural and physicochemical property space between Glide- and SVM-Agent generated molecules

**Fig. 8:**
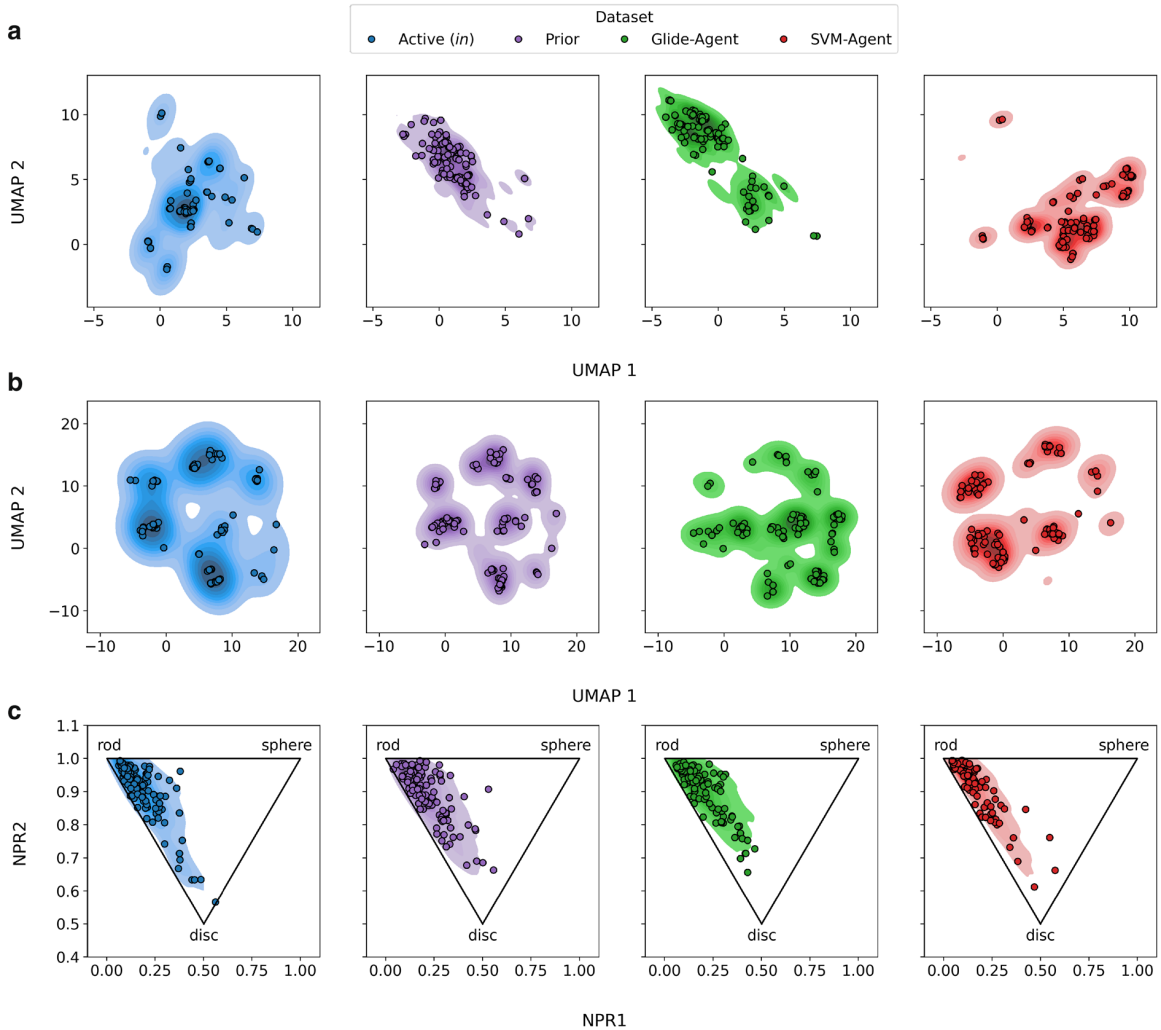
Chemical space representation of (**a**) molecular fingerprints and (**b**) physicochemical descriptors and (**c**) 3D space via moments of inertia. The plots show the calculated kernel density estimate with 100 randomly drawn samples overlayed. UMAP representation (**a**–**b**) was calculated for known active DRD2 ligands with filters applied to impose a similar chemical space, as well as the chemical structures associated with the Prior, Glide- and SVM-Agents. The Agents occupy complementary regions of topological space (**a**), physicochemical property space (**b**) and slightly 3D space (**c**) (where the Glide-Agent stretches slightly more towards spherical and the SVM-Agent slightly more towards disc shape)

To further understand the chemical differences between the molecules generated by the Glide- and SVM-Agent Uniform Manifold Approximation and Projection (UMAP) [89] was used to reduce the molecular fingerprint and physicochemical and property descriptor-based representations of chemical structures into two dimensions for visualization purposes. Furthermore, we investigate the 3D shape of molecules by looking at the normalized principal moments ratio (NPR) [90]. Figure 8 shows the two-dimensional embedded space of known DRD2 active molecules (filters applied to impose similar chemical space), as well as Prior, Glide- and SVM-Agent generated de novo molecules. When molecules are defined by their molecular fingerprints (Fig. 8a), the Glide- and SVM-Agents occupy different regions of chemical space, of which neither have significant distribution overlap with known DRD2 active molecules. The SVM-Agent de novo molecules are more distinct from the Prior molecules, albeit still restricted by nature of the optimization function and inclusion of the Prior likelihood. In Fig. 8b, where molecules are defined by physicochemical and property descriptors, the Prior and Glide-Agent generated de novo molecules occupy a complementary and more diverse area of property space than SVM-Agent molecules. By annotating this embedding, it can be seen that the clustering predominantly correlates with the number of hydrogen bond donors and number of aromatic/aliphatic rings (see Additional file 1: Figure S10). Figure 8c shows a smaller difference in the distribution of 3D shapes between the datasets, again the models show slight complementary behaviour where the Glide-Agent distribution stretches slightly more towards spherical shapes and SVM-Agent slightly more towards disk shapes, although this difference is minor. The observations seen here are similar when considering *‘all’* DRD2 actives extracted from ExCAPE-DB (see Additional file 1: Figure S11), however, the representation is compressed due to larger and more distinct molecules seen in the active set. This analysis further corroborates, in a visual manner, the chemical differences between the structure- and ligand-based approaches, and the additional physicochemical diversity obtained by the Glide-Agent, which is not biased towards the properties of known bioactive molecules. For further exploration, we refer readers to Additional file 2 that allows exploration and visualization of chemical structures associated with embedded molecules.

### Characterization of de novo ligand chemistry

In order to understand the occupation of chemical space at the end of the runs on a ligand structural level, the molecules in each dataset were clustered according to their Bemis-Murcko scaffolds [88] which resulted in more stringent clusters more akin to chemical series. When filtering out clusters with less than 10 members (i.e., smaller ‘virtual series’), the Glide-Agent set contained more clusters with better mean docking scores than all other datasets (see Additional file 1: Figure S12). More specifically, the Glide-Agent set contains 30 such clusters with a mean docking score less than the previously defined threshold of -8.5, compared to just six clusters of DRD2 actives, 22 in SVM-Agent set and zero clusters in the Prior set. In this way, the Glide-agent was able to identify chemical series that dock consistently well; something that is less frequently observed for the SVM-Agent or even known actives, and non-existent for Prior de novo molecules. This behaviour is analogous to the identification of bioactive chemical series in an experimental screening, where additional confidence is provided that the compounds identified are indeed true positive hits, as opposed to singletons, as false positives can occur due to experimental error (or, in the current case, idiosyncratic behaviour of the scoring function). Alternatively, it could be argued that the scoring function is not sensitive enough to identify subtle differences in ligand chemistry that result in inactivity, commonly referred to as activity cliffs i.e. strong nonadditivity in structure–activity relationships. However, one study investigated strong nonadditivity between matched molecular pair cycles with respective structural data [99], and identified that in 10 out of 15 possible cases there was a structural explaination, such as, complete rearrangement of binding mode or substituent interactions causing nonadditivity. Therefore, we theorize that scoring functions that take into account structural information may better account for nonadditivity than purely ligand-based ones.

**Fig. 9:**
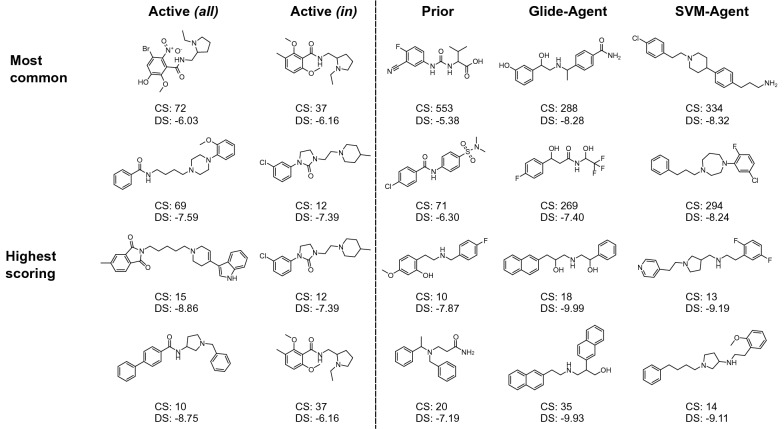
Most common and highest-scoring chemotypes of two most highly populated and the two highest-scoring clusters for each individual dataset, annotated by cluster size (CS) and mean cluster docking score (DS). The Glide- and SVM-Agent generated molecules show similar mono- or bicyclic capping of molecules as known DRD2 active molecules

Figure 9 shows the cluster centroids of the two largest and the two best-scoring clusters from each respective dataset (minimum of 10 clusters). Typical known DRD2 active molecules are ‘capped’ by mono- or bicyclic systems which are linked by an aliphatic chain that usually (but not exclusively) contains a piperidine/piperazine moiety. This chemotype is not well recapitulated by the Prior molecules as it is not optimized towards DRD2 bioactivity in any way. The Glide-Agent on the other hand learns to mostly cap the molecules with mono- or bicyclic systems, but it does not generate the piperidine/piperazine moiety in the compounds shown here. Likewise, the SVM-Agent also learns to cap the molecules in this manner, and the highest-scoring cluster centroids also contain aliphatic chains with rings in the linker, although commonly pyrrolidine and diazepane, as opposed to piperidine or piperazine. At least one protonatable nitrogen is common across most structures (from either origin), mostly located in the aliphatic linker. Somewhat concerningly, some of the example structures shown in Fig. 9 have the potential to be di-cationic. This can be undesirable from a drug discovery perspective due to low logD and thus, potential implications with high clearance and low permeability. We investigated this further, and found (see Additional file 1: Figure S13) that the distribution of formal charge (as assigned by our protocol) for the Glide-Agent closely resembles that of known DRD2 actives (predominantly + 1). In fact, the SVM-Agent is slightly shifted towards containing more di-cationic molecules (~ 30%), despite the SVM being trained on known DRD2 actives (< 10% di-cationic). Furthermore, the Glide-Agent was able to improve the docking score distribution from the Prior for all formal charges (see Additional file 1: Figure S14). Overall, we can conclude that we did not find any evidence that di-cationic molecules were preferred by the Glide-Agent due to any biases in the scoring method employed.

One crucial requirement of de novo molecules for practical use is synthetic accessability. In this work, we find that both Prior and Agent generated molecules closely inherit the *SAscore* distribution of the ZINC training dataset (see Additional file 1: Figure S15) which is likely due to the inclusion of Prior likelihood in the optimization function [7]. Despite the fact that goal-directed optimization tasks have previously led to worse syntheizability [100]. Furthermore, we don’t find the need to add proxy functions such as *SAscore* or *QED* to the optimization function (unlike recent approaches [101, 102]) due to stringent filtering of the training dataset, of which the model does not deviate too much.

### Understanding method behaviour at the ligand–protein interaction level

**Fig. 10:**
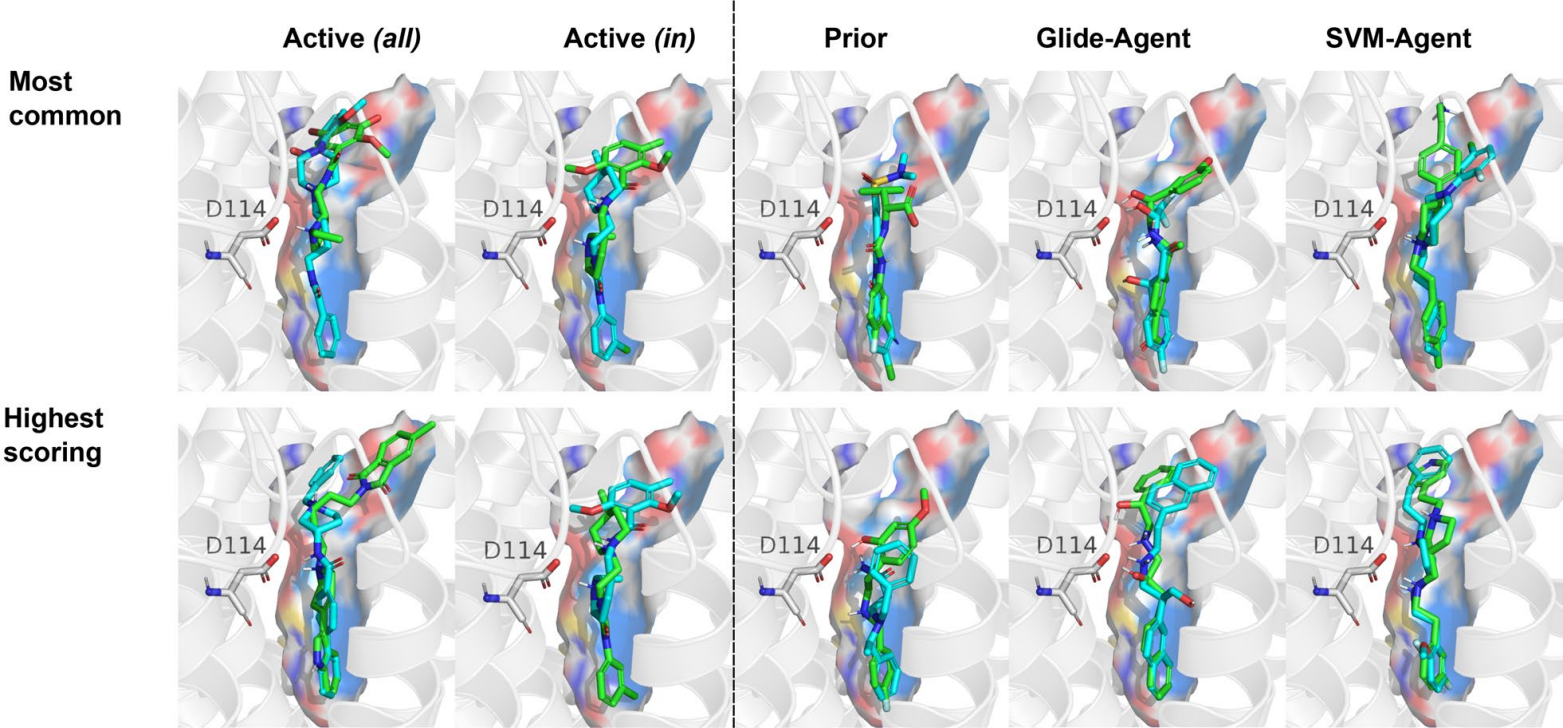
Docked pose of the cluster centroids of the two most common and highest-scoring chemotypes with DRD2. The highest-ranked ligand in both cases is displayed with sticks (green), and the second-highest ligand with lines (cyan). The Glide- and SVM-Agent examples both reproduce crucial D114^3x32^ interactions

In order to interpret the interactions formed by de novo ligands originating from the different methods also at the ligand–protein interaction level, the docked poses of the two highest-scoring and the two most common cluster centroids from Fig. 9 were generated (Fig. 10). As expected, known DRD2 ligands form a hydrogen-bond interaction with D114^3x32^, a highly conserved residue in aminergic receptors that has been shown to be crucial for ligand binding [66, 67]. This reproduction of charge interactions with D114^3x32^ can be observed in the highest-scoring molecules across all datasets, while in this instance, the Glide-Agent molecules show more distinct D114^3x32^ interaction types (e.g. also hydroxyl interactions, Fig. 10) and vectors.

**Fig. 11:**
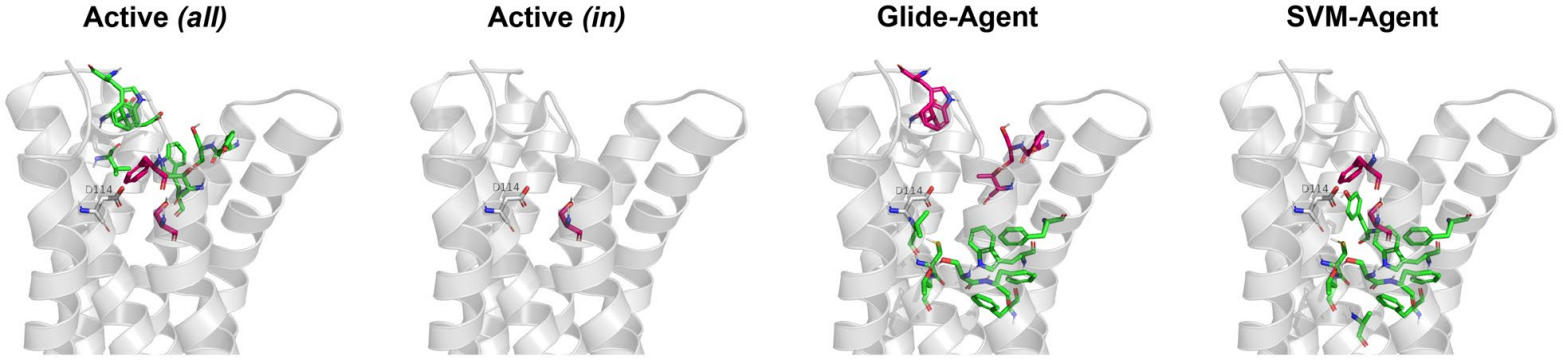
Change in the frequency of DRD2 residue interactions relative to Prior de novo molecules according to Structural Interaction Fingerprints (SIFTs). Green indicates a relative increase equal to or more than 10% than Prior molecules, while red indicates a decrease less than or equal to 10%

To understand the protein–ligand interactions present in the datasets on a broader scale, Structural Interaction Fingerprints (SIFts) [94] were calculated. Figure 11 summarises the changes in these interactions observed relative to the Prior (as a baseline) visually. All DRD2 binders extracted from ExCAPE-DB tend to form more interactions with residues located higher in the pocket (towards the extracellular surface). While the Glide-Agent molecules more often satisfy interactions deeper in the pocket and less often shallower ones (dissimilar to known DRD2 active molecules). Likewise, SVM-Agent molecules more often form interactions with residues deeper in the pocket. This is likely partially due to the restriction in molecular weight imposed by the ZINC subset used to train the Prior, which selects molecules with a molecular weight between 250 and 350 Daltons, subsequently biasing de novo molecule generation to a similar molecular weight range. Furthermore, when only considering actives with the same filters applied (i.e., molecular weight 250–350 Da) there are few residue interaction differences compared to Prior generated molecules. Surprisingly, the Glide-Agent de novo molecules have a *lower* molecular weight distribution (see Additional file 1: Figure S15), showing that in the current case smaller molecules are favourable for optimizing docking score, resulting in increased virtual ligand efficiency. This is in contrast to previous publications, which frequently found that larger molecules are favoured by many scoring functions [103, 104]. Although there is no relative change in the sum of interactions satisfied with D114^3x32^ (despite its crucial role in ligand binding), the ratio of interaction type changes between datasets. The Glide-Agent de novo dataset has a higher fraction of charged hydrogen-bonding interactions (~ 0.75) than the Prior (~ 0.4), SVM-Agent (~ 0.6) and known DRD2 actives (~ 0.4–0.5), where all other interactions are comprised of charged non-hydrogen-bonding interactions (see Additional file 1: Figure S16). In addition, charged hydrogen-bonding interactions were associated with a better docking score distribution than charged non-hydrogen-bonding interactions (see Additional file 1: Figure S17), an association which is also experimentally confirmed with higher affinity [105]. In summary, Glide-Agent optimized de novo molecules satisfy more charged hydrogen-bonding interactions with D114^3x32^ and generate lower molecular weight molecules than known DRD2 active molecules and SVM-Agent de novo molecules.

## Conclusions

In this work we integrated a generative molecular de novo algorithm with ligand–protein docking and compared the results obtained to a ligand-based scoring function. We show on a commonly used benchmark dataset for the Dopamine D2 receptor that this approach results in chemically sensible molecules, which can improve docking scores beyond that of known receptor ligands, while exhibiting increased physicochemical diversity compared to using the ligand-based scoring function. The work presented here demonstrates the use of deep generative models in settings also where no ligand data is available, or novelty is of particular interest (provided an X-ray crystal structure or a suitable homology model of the target is available). Further validation on a variety of protein targets is both required and currently ongoing. Moreover, this work only investigates the optimization of the Glide docking score and does not validate alternative structure-based scoring functions. While we expect other scoring functions to be equally optimizable, the resulting de novo chemistry may differ as a function of other forcefield implementations and/or scoring function definitions such as changes in interaction terms, for example. Preliminary analysis (data not shown) suggests that this is the case when using Smina as opposed to Glide. Future work is also intended to further investigate the impact of incorporating prior structural knowledge, such as particular water/residue interactions that can affect selectivity.

## Supplementary Information

**Additional file 1.** Supporting information, tables and figures.

**Additional file 2:** UMAP representation of the topological space occupied by known active DRD2 molecules and de novo molecules.

## Data Availability

The code and datasets supporting the conclusions of this article are included within the article (and its additional files) or is made available at https://github.com/MorganCThomas/MolScore.

## Abbreviations

ADAM: Adaptative moment estimation
DRD2: Dopamine receptor D2
FEP: Free energy perturbation
FCD: Fréchet chemnet distance
GPCR: G protein-coupled receptor
QED: Quantitative estimate of drug-likeness
QSAR: Quantitative structure–activity relationship
SAscore: Synthetic accessibility score
SELFIES: Self-referencing embedded strings
SIFt: Structure-interaction fingerprint
SMILES: Simplified molecular-input line-entry system
SVM: Support vector machine
TPSA: Topological polar surface area
UMAP: Uniform manifold approximation and projection
