# Supplementary material for: Comparison of structure- and ligand-based scoring functions for deep generative models: a GPCR case study
Source: J Cheminform. 2021 May 13;13:39. doi: 10.1186/s13321-021-00516-0 (PMC8117600; doi:10.1186/s13321-021-00516-0)
Supplement: Supplementary file 1 — Additional file 1. Supporting information, tables and figures. [file 13321_2021_516_MOESM1_ESM.pdf]

Supporting information: Comparison of structure- and ligand-based scoring functions for deep generative models: a GPCR case study

Morgan Thomas[1], Rob Smith[2], Noel M. O'Boyle[2], Chris de Graaf[2*] and Andreas Bender[1*]

[*] corresponding authors: chris.degraaf@soseiheptares.com, ab454@cam.ac.uk

[1] Centre for Molecular Informatics, Department of Chemistry, University of Cambridge, Cambridge, CB2 1EW, UK

[2] Computational Chemistry, Sosei Heptares, Steinmetz Building, Granta Park, Great Abington, Cambridge, CB21 6DG, UK

Supplementary methods

Model performance metrics

The following metrics were used to assess model performance (unless otherwise stated, RDKit was used to canonicalize SMILES):

- *Validity* is the fraction of SMILES strings that are parsed by RDKit [1], in this case; this indicates whether a SMILES string translates to a real structure.
- *Uniqueness* is the fraction of unique molecules, where non-unique molecules are defined as having canonical SMILES that match those previously sampled or in the same batch. Low *uniqueness* is indicative of a poorly behaving model that is 'stuck' in a particular region of chemical space.
- *Novelty* is the ratio of *valid, unique* canonical SMILES not present in the training dataset (ZINC subset), and low *novelty* indicates the model cannot generalize beyond training data, which is precisely the aim of *de novo* design.
- *Filters* is the ratio of *valid, unique* molecules that pass the filters applied the training dataset as implemented in the original publication [2] (i.e., not allowing charged molecules).
- *Internal diversity ($IntDiv_1$)* is one minus the average pairwise Tanimoto similarity (or Jaccard index) of all molecules, more specifically the MOSES implementation [2] calculates the Tanimoto similarity of Morgan fingerprints (r*adius*=2, *nBits*=1024) using RDKit [1]. $IntDiv_2$ is the square root of the average pairwise squared Tanimoto similarity [2]. Low *internal diversity* is an indication that a model samples from a very narrow range of chemical space.
- *Fréchet ChemNet Distance (FCD)* [3] was used to enable comparison with previous studies, which measures the mean and covariance of the penultimate layer of ChemNet [4] for two datasets. This provides a measure of distance between two datasets and has shown to take into account differences in predicted properties related to *internal diversity*, 'drug-likeness', logP and synthetic accessibility proxies [3].
- *Single nearest neighbour similarity (SNN)* is the average maximum Tanimoto similarity of a dataset to a reference dataset, more specifically the MOSES implementation [2] calculates the Tanimoto similarity of Morgan fingerprints (r*adius*=2, *nBits*=1024) using RDKit [1]. This provides a measure of on average how close the most similar molecules are between datasets.
- *Fragment similarity (Frag)* is the cosine distance between the frequency of substructures in two datasets as enumerated using BRICS fragmentation [5] in RDKit

[1]. This provides a measure of substructure distribution similarity between two datasets.

- *Scaffold similarity (Scaff)* is the cosine distance between the frequency of Bemis-Murcko scaffolds [6] in two datasets as implemented in RDKit [1]. This provides a measure of scaffold distribution similarity between two datasets.

In addition to the above metrics, we extend the performance metrics to include:

- *Scaffold diversity (ScaffDiv)* is identical to the *internal diversity,* however, calculated instead on the Morgan fingerprints (*radius*=2, *nBits*=1024) of the Bemis-Murcko scaffolds [6] using RDKit [1]. This allows further interpretation as to whether the model is generating similar scaffolds.

- *Scaffold uniqueness (Scaff uniqueness)* is the fraction of unique Bemis-Murcko scaffolds [6] within a set of *valid, unique* molecules. This extends interpretation of *uniqueness* to scaffold space, where a low number indicates the model has focussed chemical space to a focussed set of Bemis-Murcko scaffolds.

Supplementary tables

Table S1. Basic generative model metrics of the Prior, Glide-Agent (@2000 steps) and SVM-Agent (@500 steps).

**Model**	**#**	**Valid** (↑)	**Unique** (↑)	**# valid & unique** (↑)	**Novelty** (↑)	**Filters** (↑)
Random	10000	1.0	1.0	10000	0.720	0.938
Train	10000	1.0	1.0	10000	0.0	0.999
Prior	10000	0.988	1.0	9879	0.800	0.995
SVM-Agent	9979	0.990	0.897	8865	**0.995**	0.964
Glide-Agent	**9993**	**0.990**	**0.953**	**9434**	0.978	**0.967**

Table S2. Diversity metrics of the Prior, Glide-Agent (@2000 steps) and SVM-Agent (@500 steps).

**Model**	**IntDiv$_1$** (↑)	**IntDiv$_2$** (↑)	**SEDiv** (↑)	**SEDiv @1k** (↑)	**ScaffDiv** (↑)	**Scaff uniqueness** (↑)
Random	0.874	0.868	0.440	0.809	0.857	0.757
Train	0.863	0.856	0.366	0.753	0.844	0.687
Prior	0.863	0.857	0.386	0.756	0.844	0.699
SVM-Agent	0.752	0.741	0.044	0.124	0.720	0.293
Glide-Agent	**0.831**	**0.821**	**0.123**	**0.337**	**0.797**	**0.381**

Table S3. Similarity metrics of the Prior, Glide-Agent (@2000 steps) and SVM-Agent (@500 steps) to training and held out test data.

**Model**	**FCD** (↓)			**SNN** (↑)		**Frag** (↑)		**Scaff** (↑)	
	Train	Test	TestSF	Test	TestSF	Test	TestSF	Test	TestSF
Random	3.110	3.109	3.269	0.544	0.517	0.980	0.977	0.474	0.208
Train	0.124	0.129	0.660	0.645	0.584	1.000	0.998	0.865	0.000
Prior	0.133	0.138	0.643	0.614	0.565	1.000	0.998	0.850	0.076
SVM-Agent	35.975	35.952	35.977	0.504	0.492	0.502	0.499	0.040	0.005
Glide-Agent	**16.462**	**16.445**	**17.533**	**0.528**	**0.502**	**0.840**	**0.828**	**0.252**	**0.075**

Supplementary figures

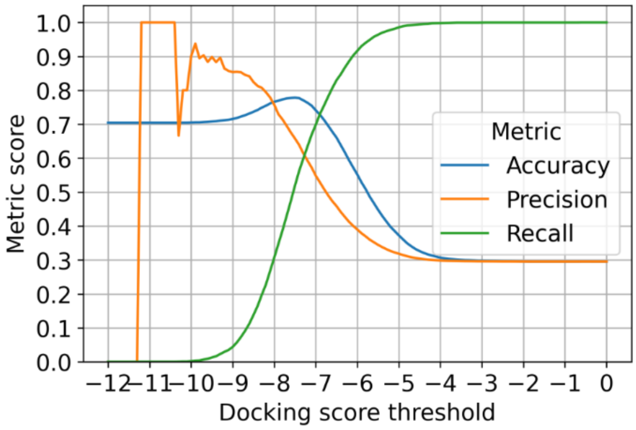

Figure S1. Retrospective performance of docking protocol as a classification problem. Retrospective performance of the Glide docking protocol on known human DRD2 active and inactive molecules extracted from ExCAPE-DB. The docking score is used as a decision threshold to predict molecules as active or inactive, and the accuracy, precision and recall are reported at a variety of docking score decision thresholds. It can be seen that a docking score threshold of -8.5 results in a precision of approximately 82%.

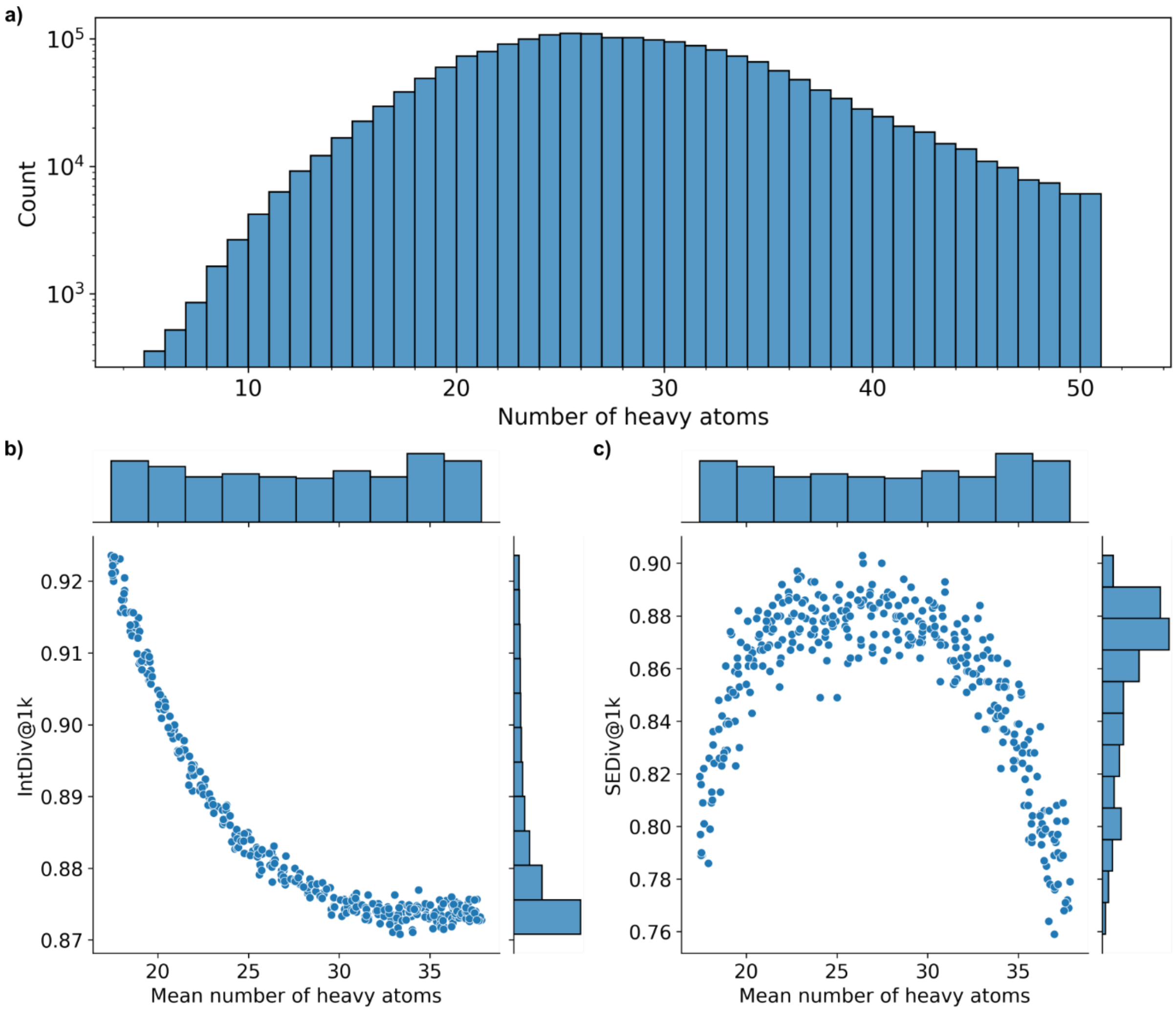

Figure S2. Investigation of the dependence of *internal diversity* on molecular size. Here we show (**a**) the count of molecules in ChEMBL28 from 5-50 heavy atoms, (**b**) the relationship between the mean number of heavy atoms and *internal diversity (IntDiv@1k)* and (**c**) the relationship between the mean number of heavy atoms and *sphere exclusion diversity (SEDiv@1k)*.

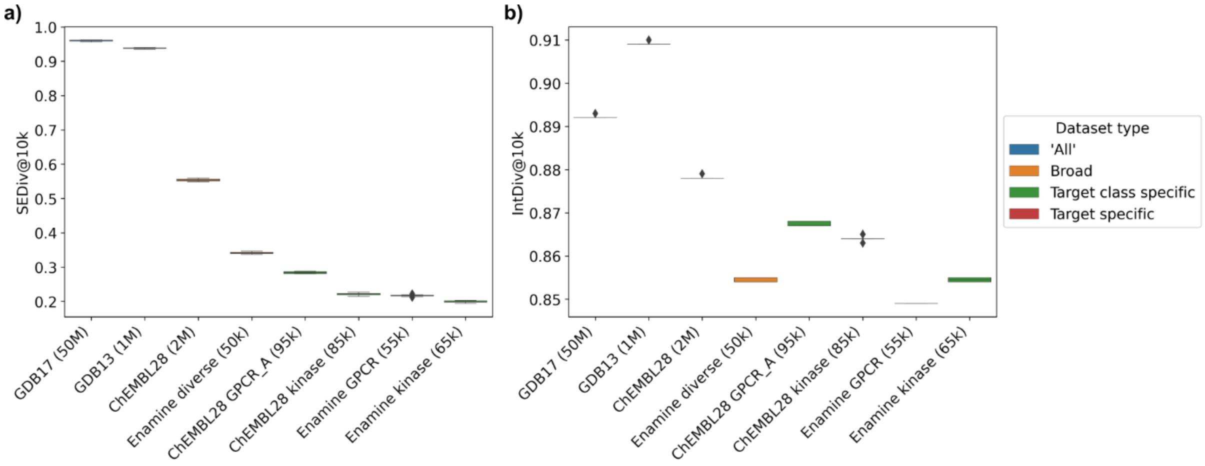

Figure S3. The measured *sphere exclusion diversity (SEDiv)* (**a**) and *internal diversity (IntDIv)* (**b**) of a randomly sampled 10,000 (@10k) subset of a variety of virtual libraries and datasets of characterised molecules with activity against particular targets belonging to a target class. *Internal diversity* measures GDB13 as more diverse than GDB17, while *sphere exclusion diversity* measures GDB17 as more diverse than GDB13 – in line with chemical intuition.

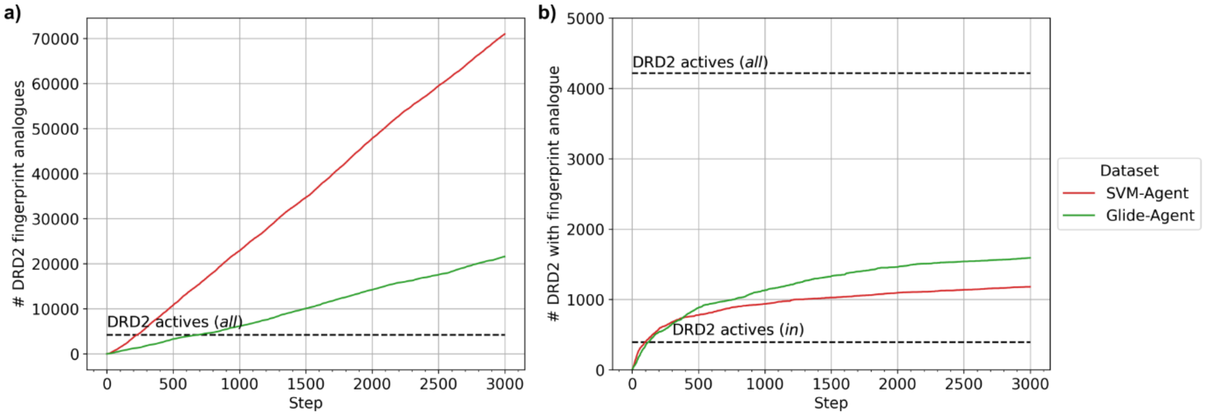

Figure S4. The cumulative number of molecular fingerprint analogues to known DRD2 active compounds (**a**) and number of known DRD2 active molecules with analogues (**b**) generated during training. The SVM-Agent generates more analogues to known DRD2 active molecules, although, the Glide-Agent generates analogues to more known DRD2 active molecules.

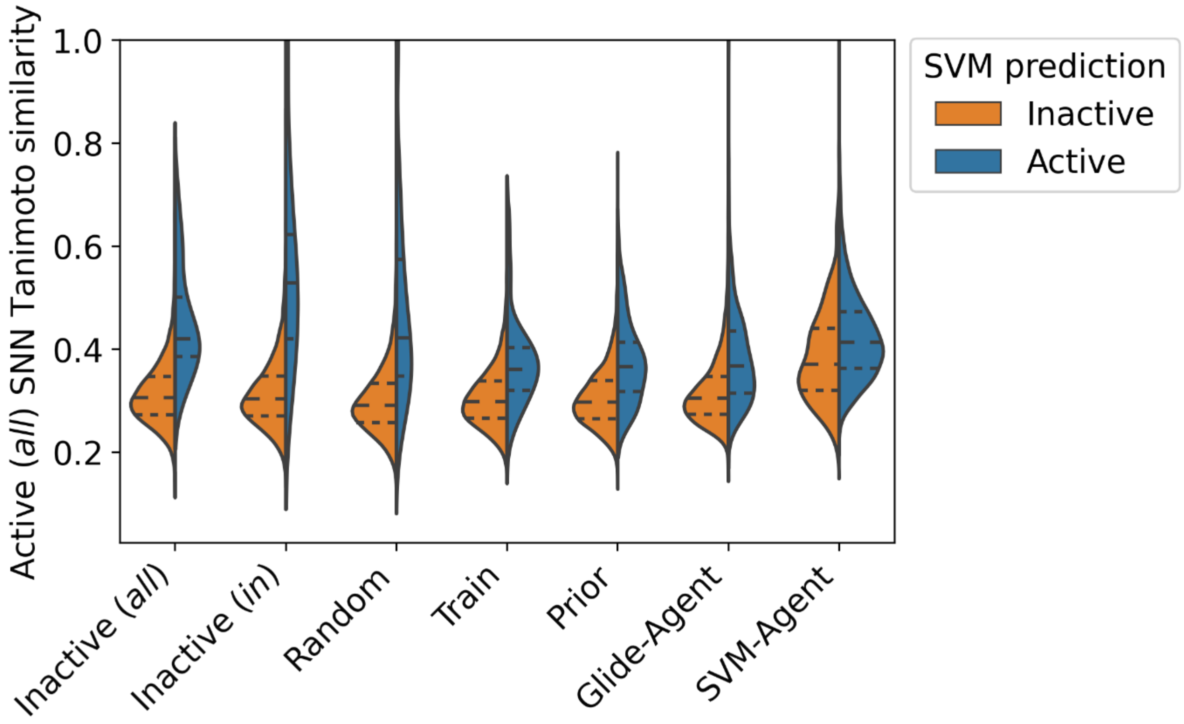

Figure S5. Single nearest neighbour (SNN) Tanimoto similarity to DRD2 active molecules, as well as predicted activity against DRD2 by the SVM. The SVM-Agent molecules have a greater mean SNN similarity to DRD2 active molecules than the Glide-Agent molecules and predicted active molecules are more similar than predicted inactive.

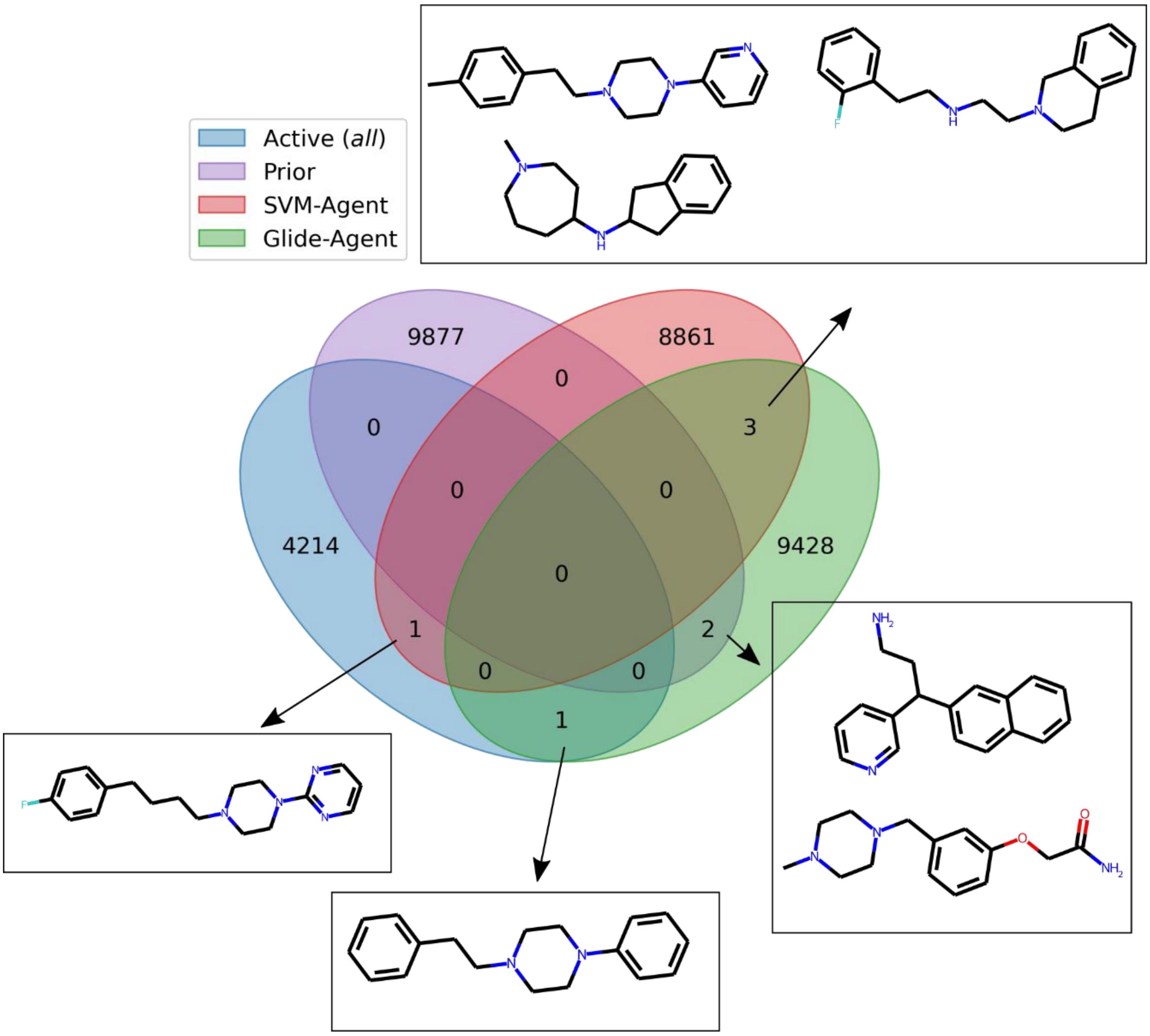

Figure S6. Overlap of *de novo* molecules to DRD2 active compounds. Each Agent only managed to recover one active molecules, and in total only shared three *de novo* molecules between them - exemplifying divergent behaviour.

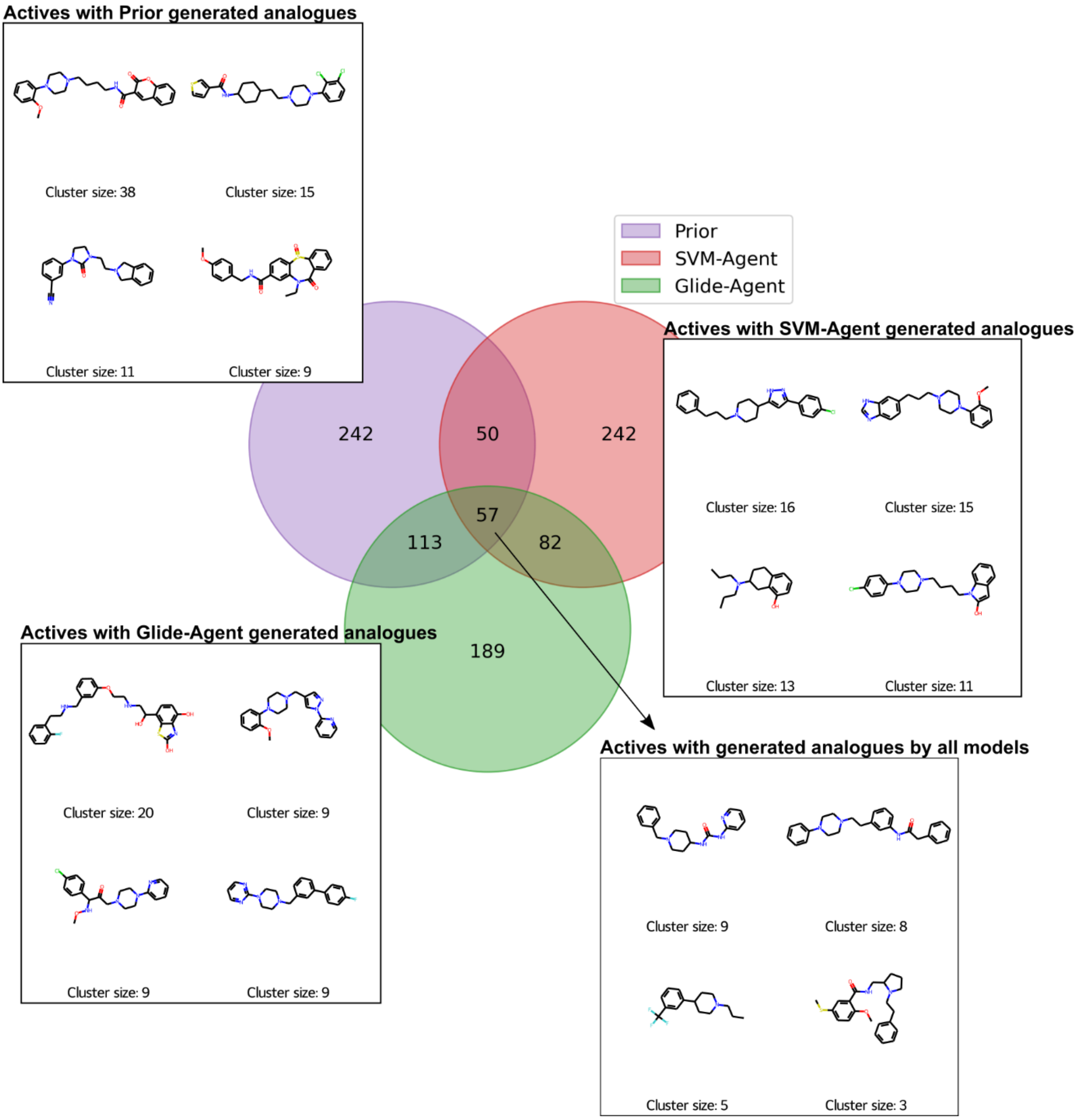

Figure S7. Overlap between Active *(all)* molecules with analogues generated by generative models. Centroids of the largest clusters are shown to represent the most common chemistry present in selected subsets. The DRD2 actives with analogues generated are different depending on the specific Agent. Suggesting these approaches are complementary to each other for discovering similar active molecules.

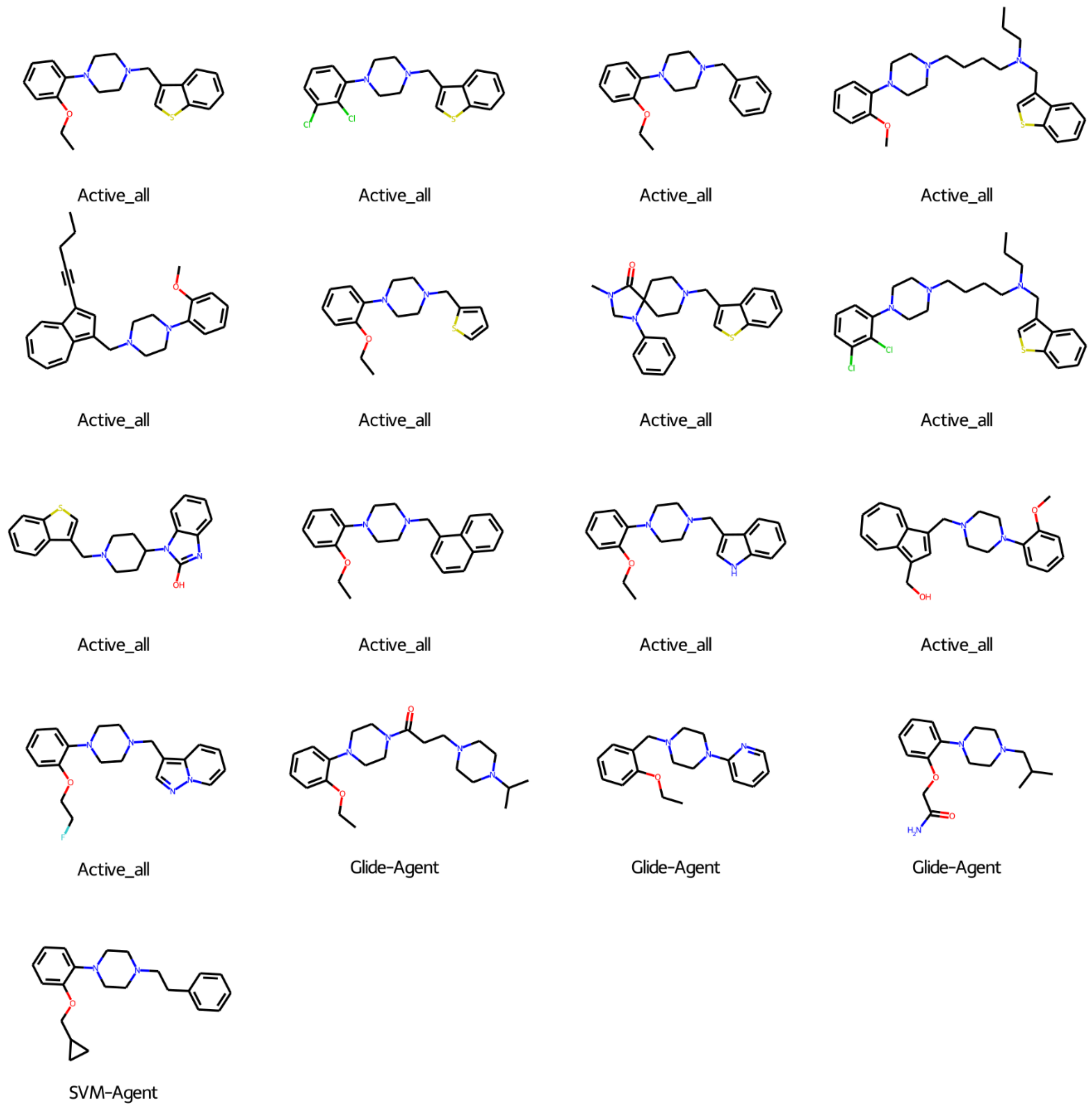

Figure S8. Example of molecule cluster when defined by whole molecule fingerprints. Molecules clustered using corresponding Morgan fingerprints and a set distance threshold of 0.65. Molecules are somewhat similar although the cluster contains different ring systems and linker lengths.

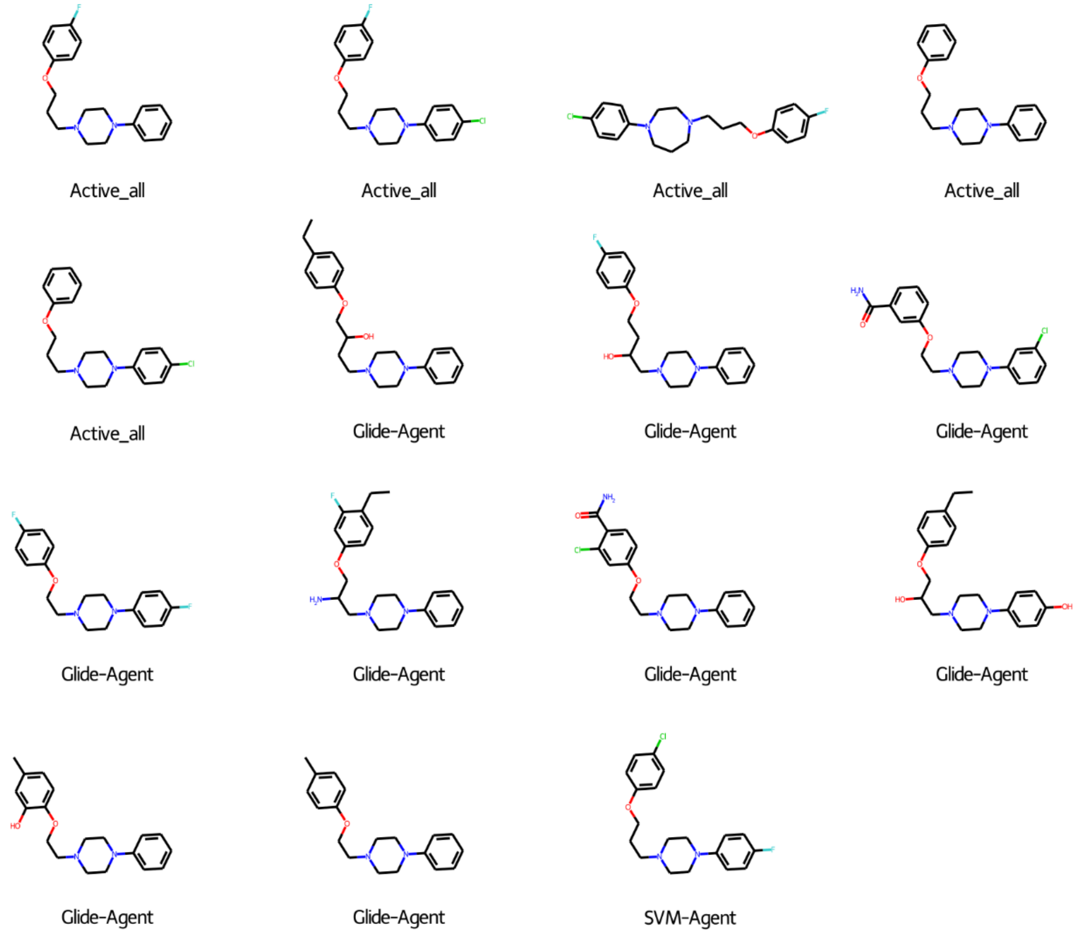

Figure S9. Example of molecule cluster when defined by Bemis-Murcko scaffold fingerprints. Molecules clustered using corresponding Morgan fingerprints of respective Bemis-Murcko scaffolds with a set distance threshold of 0.2. Molecules are very similar; the linker differs in length by two carbons or less and one central ring is one carbon larger.

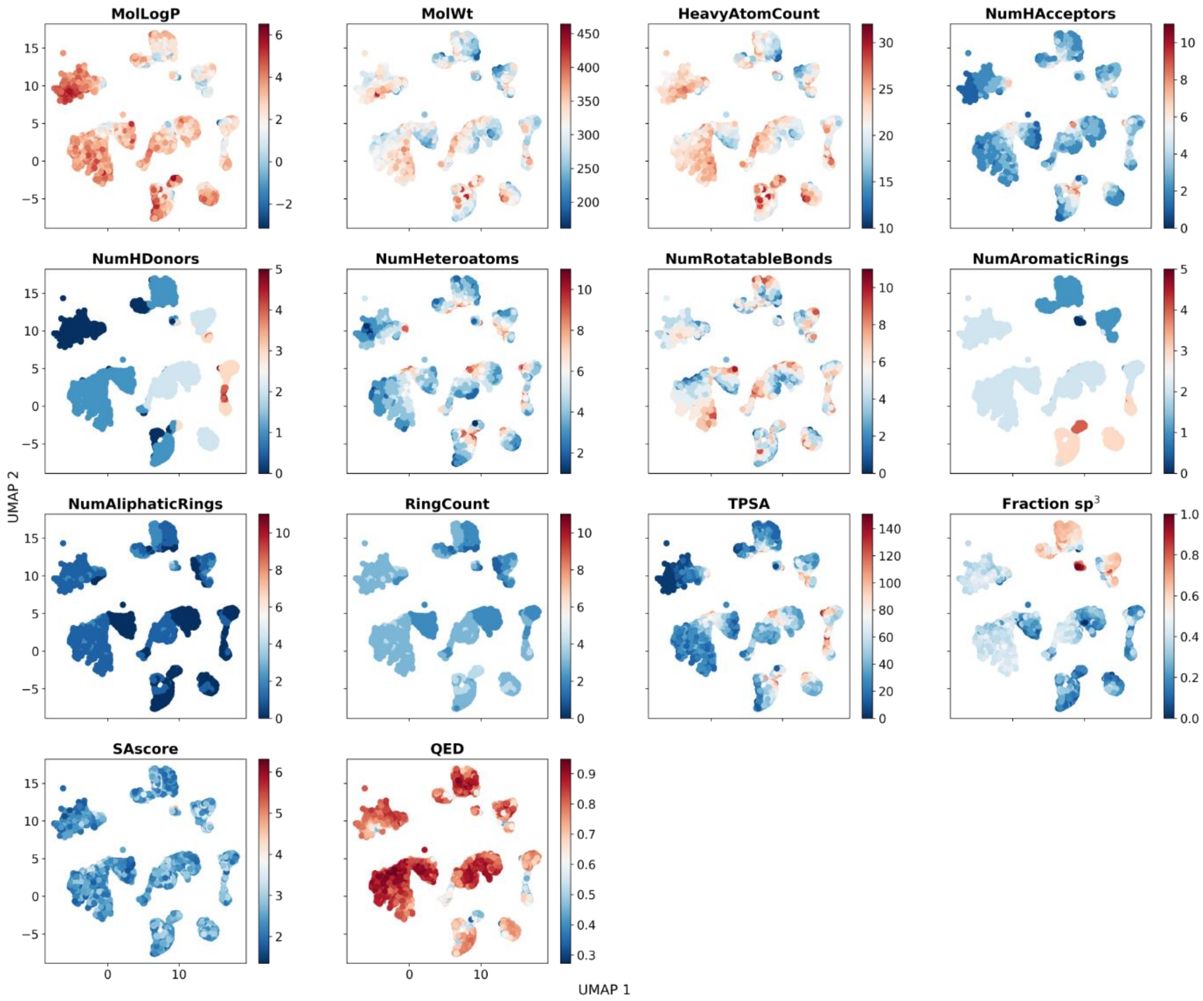

Figure S10. UMAP representation of physicochemical space as shown in Figure 8 annotated by physicochemical property descriptors used to calculate the embedding. It can be seen that the properties that most correlate with the clustering are the number of hydrogen bond donors (*NumHDonors*) and ring counts (*NumAromaticRings, NumAliphaticRings* and *RingCount*).

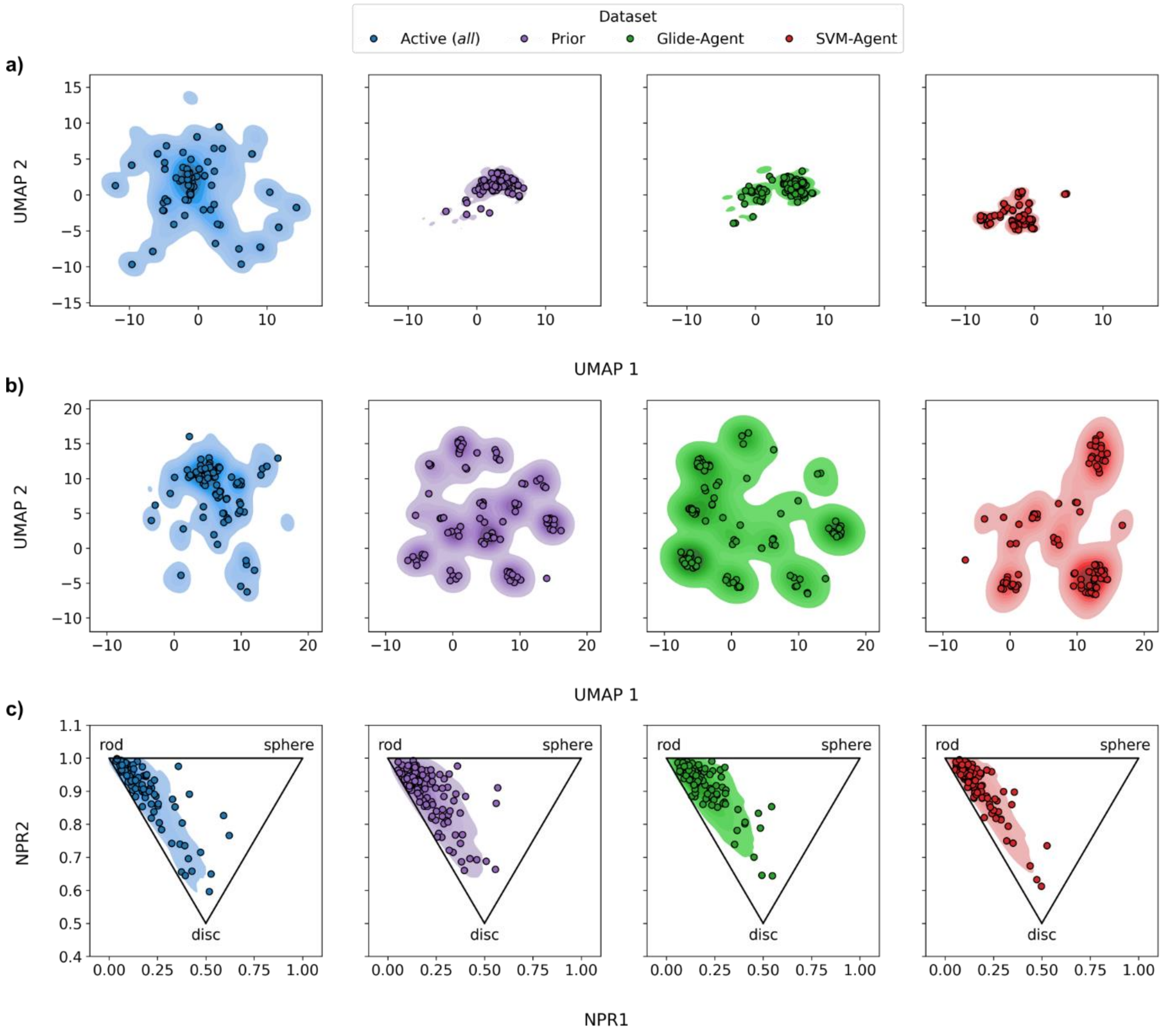

Figure S11. Chemical space representation of (**a**) molecular fingerprints and (**b**) physicochemical descriptors and (**c**) 3D space via moments of inertia. The plots show the calculated kernel density estimate with 100 randomly drawn samples overlayed. UMAP representation (**a-b**) was calculated for *all* active DRD2 ligands without filters applied, as well as the chemical structures associated with the Prior, Glide- and SVM-Agents. The Agents occupy complementary regions of topological space (**a**), physicochemical property space (**b**) and slightly 3D space (**c**). It can also be seen that the Glide-Agent better maintains the physicochemical diversity found in the Prior.

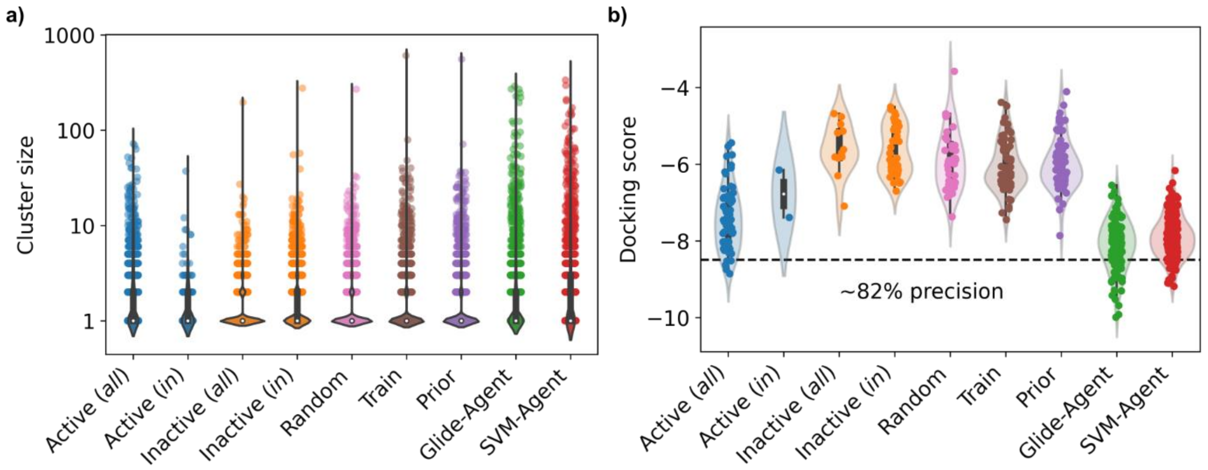

Figure S12. Size and docking score of molecular clusters. Distribution of molecular clusters (**a**) according to their cluster size and (**b**) docking score for those with a size greater than or equal to 10. Docking score reflects the mean docking score of all molecules in a cluster. The Glide-Agent contains more clusters of size 10 or more, with lower (better) mean docking scores.

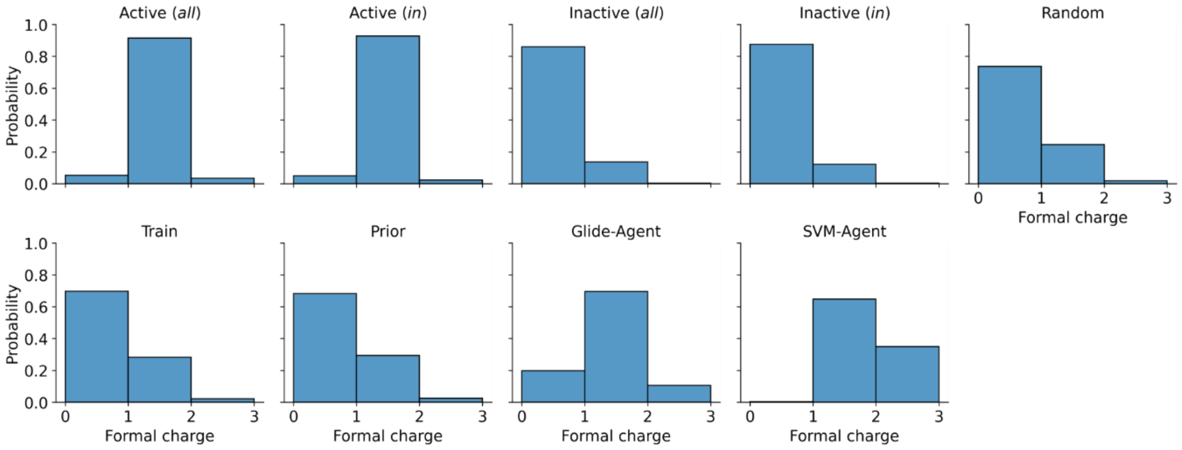

Figure S13. Formal charge distribution of datasets according to the docking protocol (i.e., protonations states possible at pH 7±1 with the best docking score). The charge distribution observed by the Prior is shifted by the Glide-Agent to closer recapitulate the distribution found in actives (all extracted from ExCAPE-DB), in fact more so than the SVM-Agent which contains more di-cationic molecules. This suggests that the docking scoring function does not over estimate charge contributions.

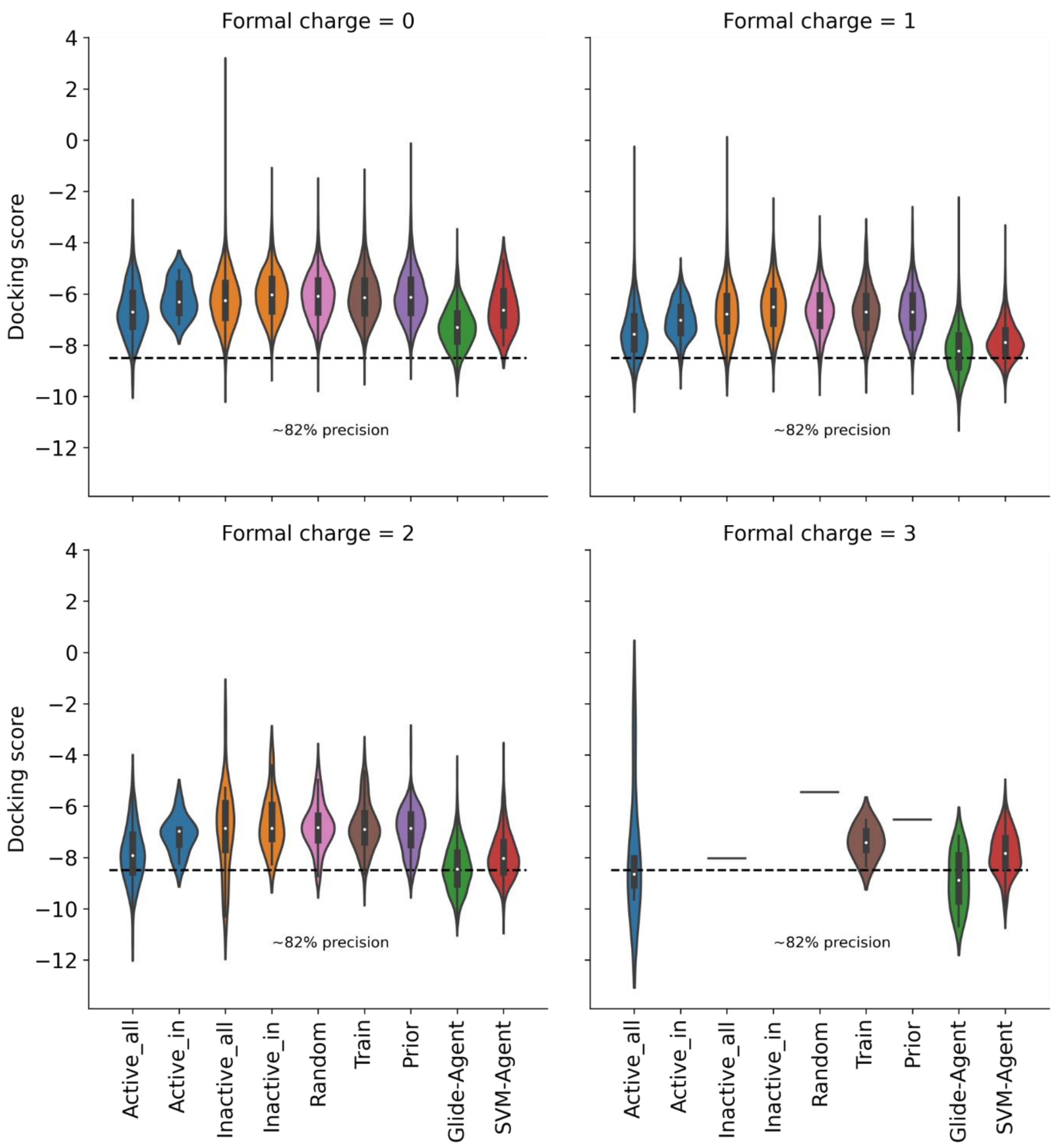

Figure S14. Docking score distribution of molecules in each dataset split by filtering certain formal charge values. The Glide-Agent provides the most or equal enrichment at all formal charge states.

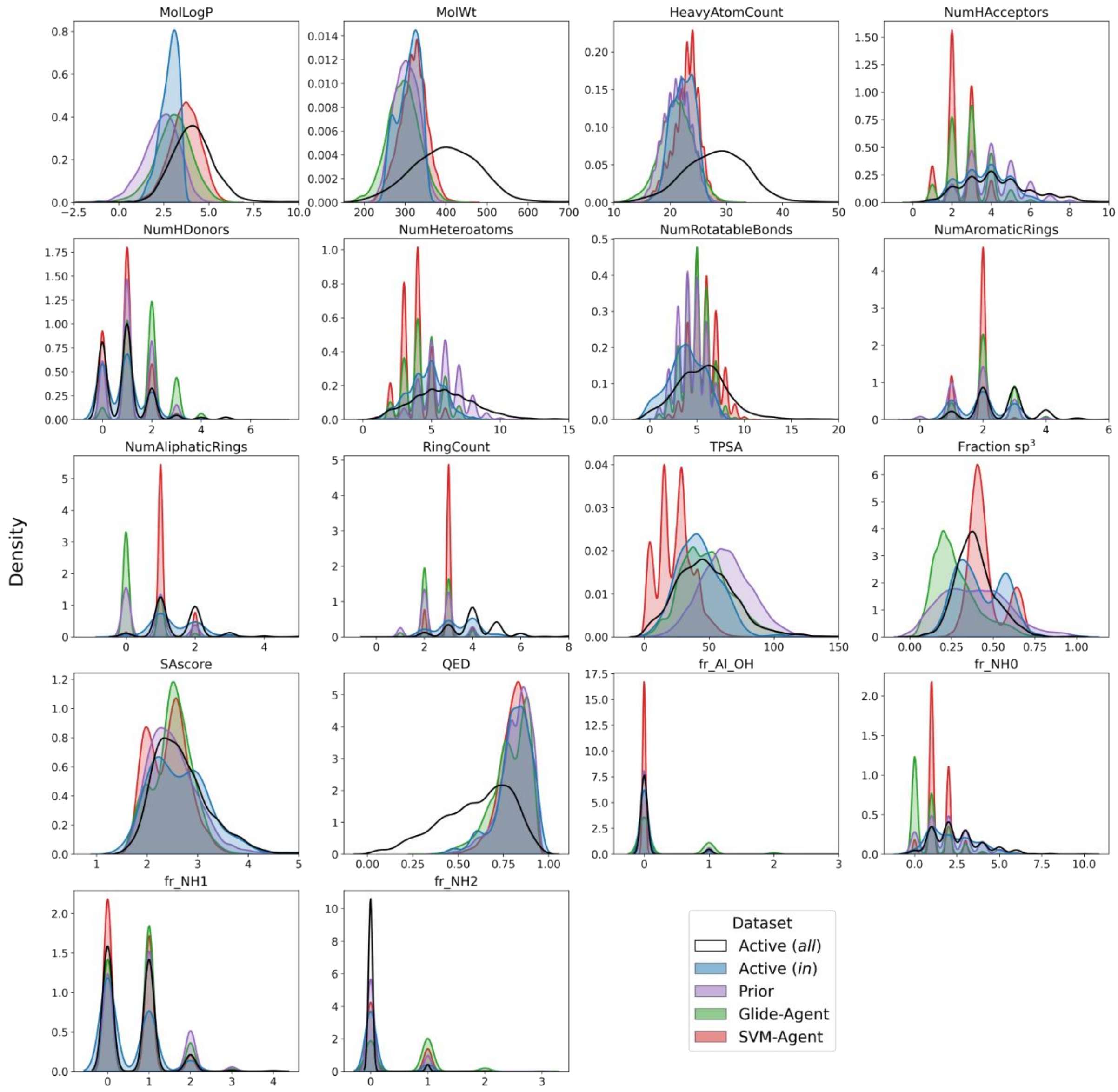

Figure S15. Kernel density estimates of physicochemical properties, SAscore, QED, number of aliphatic hydroxyl groups (fr_Al_OH) and number of tertiary, secondary and primary amines (fr_NH0, fr_NH1, fr_NH2). Of note, the Glide-Agent *MolWt* distribution diverges away from the Prior and DRD2 active molecules.

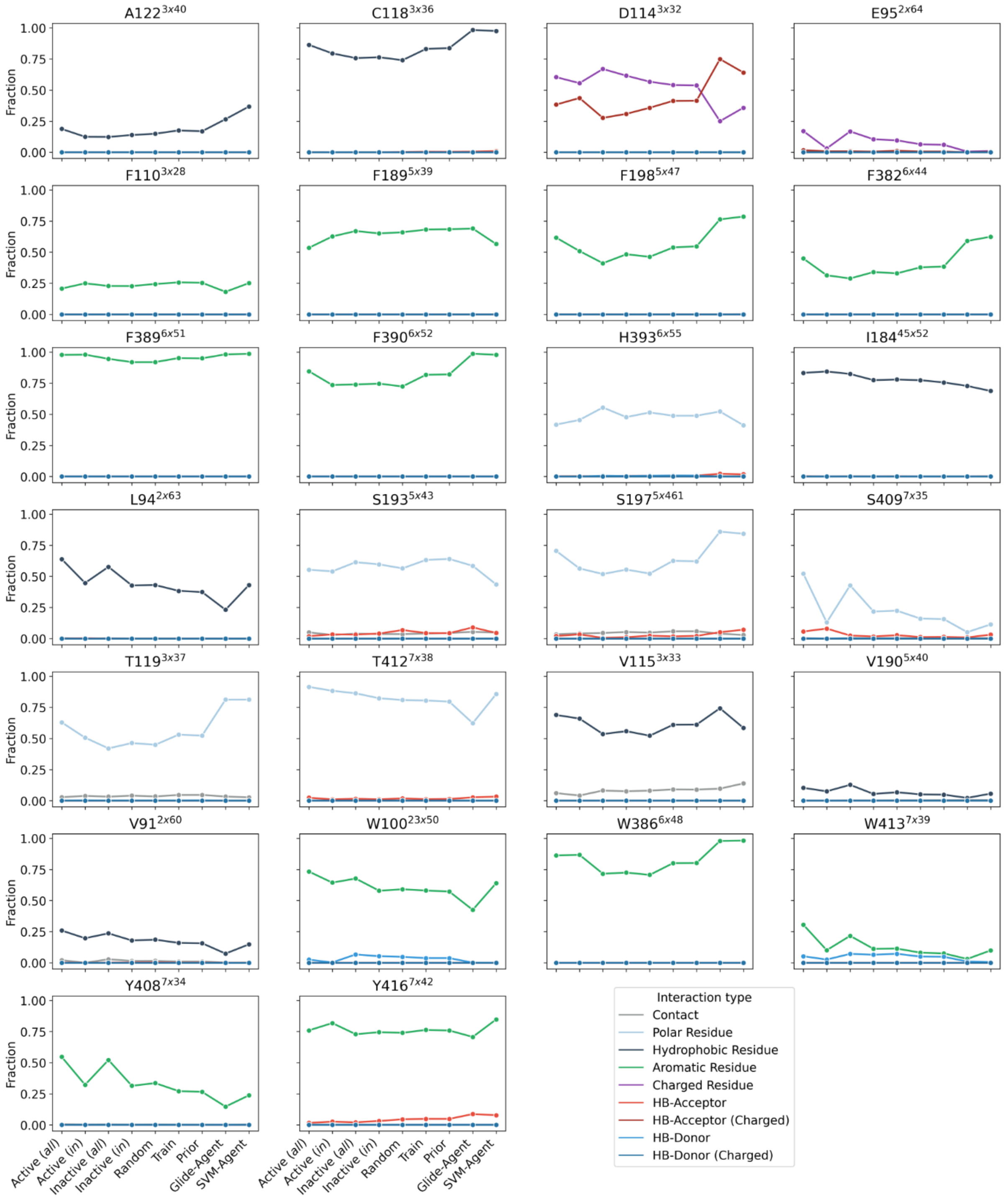

Figure S16. Fraction of Structure-Interaction Fingerprints (SIFts) satisfied by molecules from the datasets analysed according to interaction type. The ratio of interaction types against D114^{3x32} switches for the Glide- and SVM-Agent *de novo* molecules, although more so for the Glide-Agent molecules.

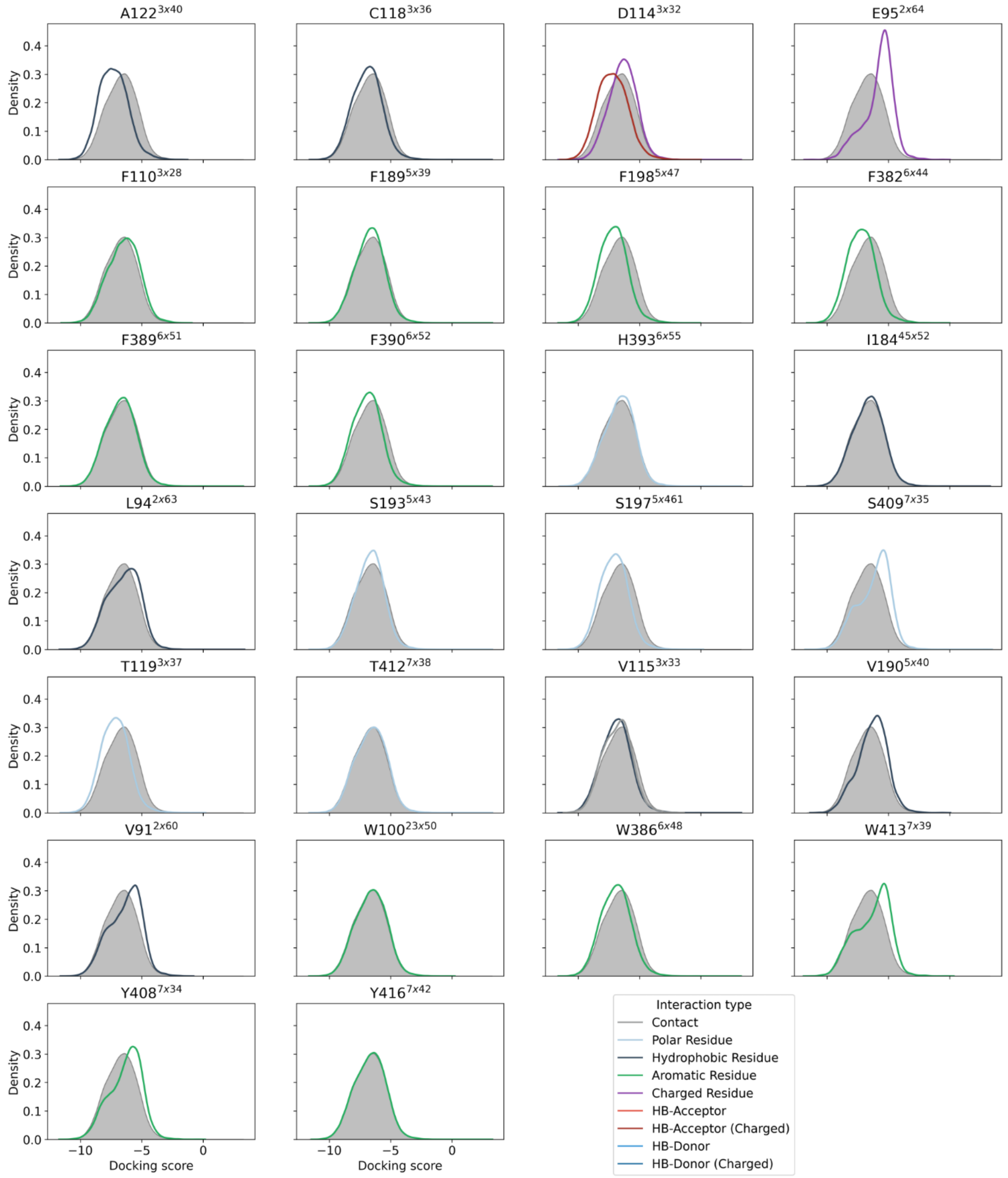

Figure S17. Association of residue interactions with docking score. Kernel density estimates of all docking scores (grey, shaded) and docking scores only when respective interactions are satisfied (coloured, unshaded). Of note, the D114^{3x32} *HB-Acceptor (Charged)* interaction is associated with better docking scores than *Charged Residue* interaction.

References

1. RDKit Open-source cheminformatics. http://www.rdkit.org

2. Polykovskiy D, Zhebrak A, Sanchez-Lengeling B, et al (2019) Molecular Sets (MOSES): A Benchmarking Platform for Molecular Generation Models. arXiv

3. Preuer K, Renz P, Unterthiner T, et al (2018) Fréchet ChemNet Distance: A Metric for Generative Models for Molecules in Drug Discovery. J Chem Inf Model 58:1736–1741. https://doi.org/10.1021/acs.jcim.8b00234

4. Mayr A, Klambauer G, Unterthiner T, et al (2018) Large-scale comparison of machine learning methods for drug target prediction on ChEMBL. Chem Sci 9:5441–5451. https://doi.org/10.1039/c8sc00148k

5. Degen J, Wegscheid-Gerlach C, Zaliani A, Rarey M (2008) On the Art of Compiling and Using “Drug-Like” Chemical Fragment Spaces. ChemMedChem 3:1503–1507. https://doi.org/10.1002/cmdc.200800178

6. Bemis GW, Murcko MA (1996) The properties of known drugs. 1. Molecular frameworks. J Med Chem 39:2887–2893. https://doi.org/10.1021/jm9602928
