# Supplementary material for: Comparison of structure- and ligand-based scoring functions for deep generative models: a GPCR case study
Source: J Cheminform. 2021 May 13;13:39. doi: 10.1186/s13321-021-00516-0 (PMC8117600; doi:10.1186/s13321-021-00516-0)
Supplement: Supplementary file 2 — Additional file 2: UMAP representation of the topological space occupied by known active DRD2 molecules and de novo molecules. [file 13321_2021_516_MOESM2_ESM.html]

0 / 0
